# Chemical-Dealloying-Derived PtPdPb-Based Multimetallic Nanoparticles: Dimethyl Ether Electrocatalysis and Fuel Cell Application

**DOI:** 10.1021/acsami.3c11003

**Published:** 2023-11-30

**Authors:** Medhanie Gebremedhin Gebru, Palaniappan Subramanian, Petr Bělský, Radhey Shyam Yadav, Itay Pitussi, Sarath Sasi, Rostislav Medlín, Jan Minar, Peter Švec, Haya Kornweitz, Alex Schechter

**Affiliations:** † Department of Chemical Science, Ariel University, 40700 Ariel, Israel; ‡ Research and Development Centre for Renewable Energy, New Technologies Research Centre (NTC), University of West Bohemia, Univerzitni, 8/2732, 301 00 Pilsen, Czech Republic; § Institute of Physics, Slovak Academy of Sciences, Dúbravská cesta 9, 845 11 Bratislava, Slovak Republic

**Keywords:** dimethyl ether, chemical dealloying, fuel cell, oxidation pathway, online mass spectrometry

## Abstract

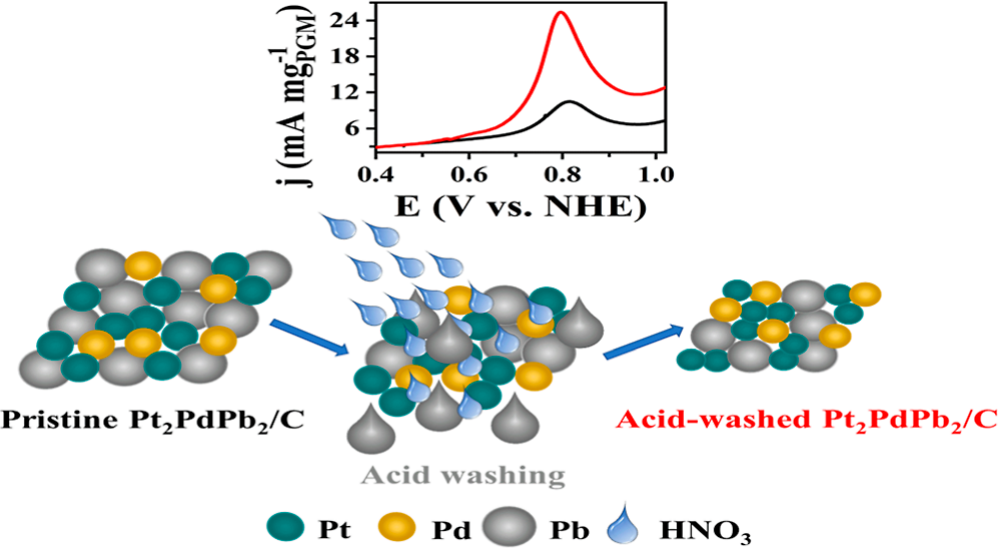

In this work, we report a novel multimetallic nanoparticle catalyst composed of Pt, Pd, and Pb and its electrochemical activity toward dimethyl ether (DME) oxidation in liquid electrolyte and polymer electrolyte fuel cells. Chemical dealloying of the catalyst with the lowest platinum-group metal (PGM) content, Pt_2_PdPb_2_/C, was conducted using HNO_3_ to tune the catalyst activity. Comprehensive characterization of the chemical-dealloying-derived catalyst nanoparticles unambiguously showed that the acid treatment removed 50% Pb from the nanoparticles with an insignificant effect on the PGM metals and led to the formation of smaller-sized nanoparticles. Electrochemical studies showed that Pb dissolution led to structural changes in the original catalysts. Chemical-dealloying-derived catalyst nanoparticles made of multiple phases (Pt, Pt_3_Pb, PtPb) provided one of the highest PGM-normalized power densities of 118 mW mg_PGM_^–1^ in a single direct DME fuel cell operated at low anode catalyst loading (1 mg_PGM_ cm^–2^) at 70 °C. A possible DME oxidation pathway for these multimetallic catalysts was proposed based on an online mass spectrometry study and the analysis of the reaction products.

## 1 Introduction

Dimethyl ether (DME)-fueled polymer electrolyte membrane fuel cells (PEMFCs) are of growing interest owing to the various advantages that DME could offer as compared to those with hydrogen and widely investigated liquid fuels, including methanol (MeOH) and ethanol.^1^ DME has a higher volumetric energy density (20 MJ/L) than that of hydrogen (4.5 MJ/L). Its ease of storage and transportation overcomes the high-pressure requirement of hydrogen as DME can be liquified under a relatively low pressure of 0.5 MPa.^2,3^ Furthermore, DME is regarded as a renewable fuel because it can be produced on a large scale from a wide range of abundantly available feedstocks, such as agricultural residues, animal waste, municipal wastes, sewage sludge, and industrial CO_2_ emissions. A direct DME fuel cell (DDMEFC) can provide 1.18 V, which is comparable to that by a direct methanol fuel cell (DMFC) (1.21 V).^4^ Compared to methanol, DME has lower toxicity (Lethal dose—LC50 measured in rats is 8.20 g kg^–1^ for DME vs 5.63 g kg^–1^ for methanol) and a lower Nafion membrane crossover from the anode to cathode owing to its lower dipole moment, 1.30 vs 1.69 D.^5^ The oxidation of DME proceeds as follows (eqs 1–3)
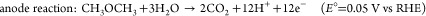
1
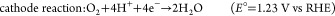
2
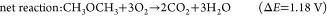
3

Complete oxidation of the DME releases 12 electrons (twice that of methanol) indicating the relatively higher energy density of DME than that of methanol (8.2 vs 6.1 kW h kg^–1^, respectively).^6^ Unlike ethanol, the absence of a C–C bond in DME allows facile oxidation to CO_2_ because of the reduction in the activation energy associated with the C–C bond scission.^7^

Despite the aforementioned benefits of renewable DME fuel, the use of DME as a fuel in low-temperature fuel cells has been limited because of the absence of low-cost and high-activity electrocatalysts. Pt-alloyed metal catalysts have been tested for their activity toward DME electro-oxidation.^4,8,9^ To date, SnO_2_-supported catalysts (PtPd/SnO_2_/C) followed by ternary metal (PtRuPd/C) catalysts have been shown to provide the highest mass-normalized peak power densities of 90 and 33 mW mg_PGM_^–1^, respectively.^4,10^ Transition metals with oxophilic properties, such as Sn, Ru, Mo, Cr, Ni, Co, and W, when alloyed with Pt-group metals (PGMs) are known to enhance the activity of noble metals (Pt or Pd) by the electronic effect^5,11^ and bifunctional mechanism.^12^ In the former effect, the transfer of electrons from Pt to the oxophilic metals alters the electronic properties of Pt, leading to the removal of CO adsorbed on the Pt surface.^13^ As suggested in a bifunctional mechanism, these metals could provide OH_ads_ and surface oxides by water activation at a lower potential to oxidize the surface-adsorbed poisoning species (CHO_ads_, −CO_ads_) formed during DME oxidation.^14^ Despite these promising improvements, the power output from DDMEFC is still significantly lower than that of hydrogen-fed PEMFCs.^15,16^

The rational design of an efficient DME oxidation catalyst requires sufficient knowledge of DME electro-oxidation pathways. Experimental and theoretical studies have reported different DME electro-oxidation pathways for different metal catalysts.^11,17^ Kerangueven et al.,^8^ reported that DME oxidation on Pt and PtM (M = Ru or Sn) starts with the adsorption and hydrolysis of DME leading to the formation of CH_2_OH_ads_ and CH_3_OH. CH_2_OH_ads_ then undergoes sequential dehydrogenation to CO_ads_ which is subsequently oxidized to CO_2_ by reacting with OH_ads_ formed by water activation. CH_3_OH is oxidized to CO_2_ via the well-known 6-electron pathway.^18^ Kashyap et al.,^10^ proposed that surface-adsorbed DME split into CH_2_OH_ads_/CHO_ads_ and CH_3_OH in the presence of H_2_O. The adsorbed intermediate (CH_2_OH_ads_/CHO_ads_) then oxidizes directly to CO_2_ or forms formic acid, which oxidizes to CO_2_ in either direct or indirect pathways at higher potentials (>0.5 V). The inconsistency between the theoretical and proposed experimental reaction pathways for different catalysts suggests that the reaction mechanism is not well understood and varies with the catalyst.

Similar to Sn and Ru in the best ternary catalyst, PtPdM (M = Sn or Ru) for DME oxidation, Pb has a stronger oxophilic character than that of Ru and is considered to promote both CO oxidation and modification of the electronic structure of Pt, resulting in superior electrocatalytic activity toward methanol oxidation.^19−22^ In the context of large-scale catalyst production and economics, Pb has been identified as an element of crustal abundance with promising potential for renewable energy technology applications.^23^ Pb is much cheaper than the widely used oxophilic metals Sn, Ru, and Bi (Table S1). To the best of our knowledge, no previous report has investigated Pb-based ternary metal catalysts for DME or other hydrocarbon electro-oxidation. In the present study, a new Vulcan XC72 carbon-supported ternary metal catalyst, Pt_*x*_Pd_*y*_Pb_*z*_/C, with varying *x*, *y*, and *z* ratios was synthesized for the electro-oxidation of DME. Research studies were conducted on a catalyst composition that contained higher initial Pb content, Pt_2_PdPb_2_/C, to attain the desired low Pt content in the nanocatalyst. We then postulated that the performance of the selected Pb-rich PtPdPb-based catalyst could be further improved by leaching out some of the inactive Pb by the HNO_3_ treatment. Detailed characterization of the catalyst before and after acid treatment was conducted by X-ray diffraction, small-angle X-ray scattering, high-resolution transmission electron microscopy, X-ray photoelectron spectroscopy (XPS), and elemental analysis. Moreover, the reaction intermediates of a DME gas-fed fuel cell were studied using online mass spectrometry, and a possible DME oxidation pathway on a new multimetallic catalyst was proposed.

## 2 Experimental Section

### 2.1 Materials

Potassium tetrachloroplatinate (K_2_PtCl_4_) (99.9%) and palladium chloride (PdCl_2_) (99.9%) were purchased from Strem Chemicals. Sulfuric acid (95–97%) and lead(II) nitrate (Pb(NO_3_)_2_) (99%) were obtained from Merck. DME was procured from Sigma-Aldrich. Toray carbon paper and Teflonized carbon cloth were purchased from FuelCellStores. Distilled water was used throughout the experiment.

### 2.2 PtPdPb-Based Catalyst Synthesis

A modified polyol method from a previous report^24^ was used to synthesize the PtPdPb-based ternary metal catalysts. The required amounts of metal salt precursors, K_2_PtCl_4_, PdCl_2_, and Pb(NO_3_)_2_, were separately dissolved in ethylene glycol (at a concentration of 4 mg mL^–1^) by vigorous stirring. The solutions were then mixed and stirred for 15 min in a round-bottom flask. Vulcan XC72 (30 wt % of metals) dispersed in ethylene glycol (4 mg mL^–1^) was added to the mixture and stirred for an additional 45 min. The pH of the mixture was adjusted to 12 by adding NaOH dissolved in ethylene glycol. The solution was heated to 100 °C for 1 h, followed by reflux heating at 180–200 °C for 2 h, while stirring. The obtained dark-black precipitate was washed with a 3:1 acetone/water mixture until a clean filtrate was obtained, and the catalyst was oven-dried overnight at 60 °C.

### 2.3 Physical Characterization

The atomic ratios of the metals were analyzed using inductively coupled plasma-optical emission spectrometry (ICP-OES 720, Varian) and scanning transmission electron microscopy coupled with energy-dispersive X-ray spectroscopy (STEM-EDS) (Super-X, Thermo Fischer Scientific). The crystal properties and phases present were analyzed by X-ray diffraction (XRD) (SmartLab, Rigaku). The size distributions of the nanoparticles were investigated by small-angle X-ray scattering (SAXS) using a Kratky-type instrument with a microfocus X-ray source with a copper anode (SAXSess mc^2^, Anton Paar GmbH), and the samples were measured as a powder glued in between two pieces of Scotch tape. The obtained azimuthally symmetric 2D scattering patterns were converted to 1D radial scattering profiles by azimuthal averaging using the software supplied with the instrument. These 1D profiles were further processed and evaluated using the Irena software package for small-angle scattering analysis.^25^ The normalized volume-weighted particle size distribution (PSD) was obtained by fitting the experimental SAXS profiles with the size distribution tool of Irena software (using the spheroid particle shape and interior-point gradient/total non-negative least squares (IPG/TNNLS)method). XPS measurements were carried out in an ultrahigh vacuum chamber with a base pressure of 1.8 × 10^–10^ mbar using a commercial energy analyzer (Phoibos 150, Specs GmbH) and a nonmonochromatic Mg Kα laboratory X-ray source with an energy of 1.254 keV. The measurements were recorded in large-area lens mode with a pass energy of 20 eV. The samples were mounted on a Mo sample plate using double-sided carbon tape for the measurements.

High-angle annular dark-field-STEM (HAADF-STEM) and STEM-EDS measurements were performed using a probe-corrected Titan Themis 300 (Thermo Fisher Scientific) microscope in STEM mode operated at 200 kV with a convergence angle of 17.5 mrad. The probe current was adjusted to ∼65 pA to minimize the beam damage. The microscope was equipped with a 4-quadrant windowless EDS detector system, (Super-X, Bruker/Thermo Fisher Scientific) and an electron energy loss psectrometer (Enfinium 977, Gatan). The transmitted electron detection system consisted of three on-axis centered annular detectors (HAADF, ADF, and annular bright-field) with collection angles of 200–101, 95–24, and 20–13 mrad, respectively. The EDS elemental concentrations were calculated in the Velox sw suite (Thermo Fisher Scientific) using the standard Cliff-Lorimer (*K*-factor) quantification method. Prior to calculations of the resulting elemental concentrations of Pd, Pt, and Pb, any eventual signal contributions from C and O were deconvoluted from the EDS spectra. STEM-EDS chemical maps were produced by integrating the background subtracted intensity of the C (Ka), Pd (La), Pt (La), and Pt (La) absorption peaks, respectively. The elemental maps show the net integrated counts according to the background corrected and fitted models. High-resolution transmission microscopy (HR-TEM) together with selected area electron diffraction (SAED) and size distribution measurements from micrographs were obtained using a JEOL JEM 2200FS. The samples were dropped onto holey carbon-coated copper grids, dried, and measured. The surface morphology of the electrodes coated with the catalyst was studied using a high-resolution SEM (HR-SEM) (MAIA3 Triglav, Tescan).

### 2.4 Electrochemical Measurement

The activities of the synthesized catalysts toward DME oxidation were evaluated using a conventional three-electrode cell. A catalyst ink was prepared by dispersing 2.5 mg of Pt_*x*_Pd_*y*_Pb_*z*_/C and 15 μL of Nafion (5% in an isopropanol–water solution, Ion power Inc.) (20 wt % of the catalyst powder) in a mixture of isopropanol and water (3:1) solvent by use of sonication. An aliquot from this ink was then drop-coated on glassy carbon of 5 mm diameter such that the catalyst loading was 70 μg cm^–2^. Catalyst-loaded glassy carbon was used as the working electrode. Ag/AgCl (Metrohm, 3 M KCl) and a graphite rod were used as the reference and counter electrodes, respectively. PtPdPb-based catalysts were activated by voltammetric potential cycling in a N_2_-saturated cell at a scan rate of 100 mV s^–1^ until a stable voltammogram was obtained. The activity of the catalysts toward DME oxidation was tested in a DME-saturated acidic electrolyte at 10 mV s^–1^.

The HNO_3_ treatment of Pt_2_PdPb_2_/C was performed by dipping the catalyst-coated glassy carbon disk electrode (PINE instruments) in HNO_3_ solutions with varying concentrations for 1 h to investigate the effect of acid treatment on the activity of the catalyst toward DME oxidation. The selection of a 1 h treatment time was based on the observation that no significant improvement in the catalyst activity was observed, and the metal of interest was not leached further beyond 1 h, as will be presented in the later sections of this manuscript. The effect of temperature on the DME electro-oxidation activity of the catalyst was studied by heating the electrochemical cell to a predefined temperature using a heating mantle.

The dissolution of the metals from the Pt_2_PdPb_2_/C catalyst when in contact with a HNO_3_ solution was evaluated using a catalyst-coated quartz crystal microbalance (QCM) in a Teflon cell containing HNO_3_. An aliquot from the diluted catalyst ink prepared as described above was drop-coated on a QCM electrode (catalyst loading: 3 ng cm^–2^) of 5 mm diameter. Measurements to record the change in the mass on the QCM electrode were started 5 min after the addition of HNO_3_ to the Teflon cell. The observed frequency change (Δ*f*) was correlated with the approximate change in mass (Δ*m*) using Sauerbrey eq 4.^26^
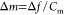
4where *C*_m_ is the microbalance sensitivity factor, whose value depends on the resonance frequency (*f*_0_), shear modulus (μ_q_), and density (ρ_q_) of the QCM, as shown in eq 5.
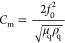
5

### 2.5 Catalyst-Coated Membrane Preparation and Fuel Cell Testing

The catalyst inks required for the preparation of the catalyst-coated membrane were prepared in a similar manner as that for the three-electrode cell, with the only difference being the addition of Teflon (10 wt % of the catalyst powder) for the cathode ink. Acid-washed Pt_2_PdPb_2_/C and Pt/C as anode and cathode catalysts, respectively, were sprayed using an ultrasonic spray-coating system (SimCoat, Sono-Tek) onto a 4 cm^2^ Nafion 212 membrane (50.8 μm) firmly held on a vacuum hot plate at 70 °C. Acid treatment of the anode catalyst was performed by stirring the powder in 0.6 M HNO_3_ for 1 h (an acid treatment procedure presumed to match the three-electrode measurement) followed by acetone washing and drying. The anode and cathode loadings were maintained at 1.0 and 2.7 mg_PGM_ cm^–2^, respectively. The prepared catalyst-coated membrane was sandwiched between Toray paper and Teflonized carbon cloth as the anode and cathode back-matter, respectively, and mounted into a commercial single-cell fuel cell (Scribner Associates Inc.). A fuel cell test station (850 g Scribner Associates Inc.) was used to control the electrode potentials and gas working conditions. Humidified anode (H_2_ or DME) and cathode (air) gases were supplied at 40 and 350 mL min^–1^, respectively. The cell and bottle temperatures were both maintained at 70 °C to ensure a relative humidity of 100%. The cell was initially conditioned by holding the anode potential at 0.2 V for 20 min followed by flowing reactant gases for 20 min to stabilize the open-circuit potential (*E*_OC_). A polarization curve was then recorded between *E*_OC_ and 50 mV repeatedly by holding the potential for 10 s for every 20 mV step to prevent fluctuation of the current.

### 2.6 Online Mass Spectrometry Experiments

DME oxidation intermediates were detected using an online mass spectrometer (HPR-20, Hiden Analytical) equipped with a crossbeam ion source (100 eV) and a Faraday cup detector. The anode exhaust of a fuel cell operating in a half cell (humidified DME and H_2_ flowing through the anode and cathode at 5 and 50 mL min^–1^, respectively) was connected to an online mass spectrometer via a capillary column heated at 160 °C. The H_2_ on Pt/C (cathode) was used as the reference electrode. The stabilization of the background spectra of the detected DME oxidation intermediates was performed at the *E*_OC_. The intermediates formed were then detected while applying constant potentials (potentiostatically) and scanning at 2 mV s^–1^ (potentiodyanmically) between 0 and 1.2 V (vs *E*_OC_).

### 2.7 Density Functional Theory Computational Details

Density functional theory (DFT) calculations were performed using the Vienna Ab-initio Simulation Program (VASP) using periodic boundary conditions.^27,28^ In all calculations, the projected augmented wave (PAW) method was used to describe the electron–ion interactions,^29^ and the generalized gradient approximation (GGA) of Perdew, Burke, and Ernzerhof (PBE)^30^ was used for the semilocal exchange-correlation functional. It was found that it is sufficient to use a cutoff energy of 500 eV and a 7 × 7 × 1 Monkhosrt *k*-point mesh for Brillouin zone sampling to obtain reproducible results.^31^ The convergence criteria for geometry relaxation were 1 × 10^–4^ eV for the total energy and 1 × 10^–4^ eV/Å for the forces. In all cases, the ion positions were relaxed, whereas the cell volume and shape remained constant. In all the calculations, van der Waals (VDW) corrections were applied using the DFT-D2 approach proposed by Grimme^32,33^ and implemented in VASP by Bučko.^34^ VASPsol^35^ was used to evaluate the solvent effects. Zero-point energy (ZPE) corrections were calculated and included in all of the Gibbs free energy values. Each slab consisted of five metallic layers and a vacuum layer of 16 Å. The Pt_2_PdPb_2_ slab contains 16 Pt atoms, 8 Pd atoms, and 12 Pb atoms yielding a surface of 75.39 Å^2^, and the Pt_2_PdPb slab contains 40 Pt, 20 Pd, and 20 Pb atoms, yielding a surface of 114.05 Å^2^. AFLOW software^36^ was used to construct the supercell.

The adsorption energy, *E*_ads_, of a molecule on a slab is defined in eq 6
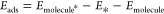
6where molecule*, *E*_molecule*_, E_*_, and *E*_molecule_ are the adsorbed molecules, total energy of the adsorbed molecule, total energy of the metallic slab, and total energy of the particle in the aqueous phase, respectively.

The reaction energy was calculated using eq 7
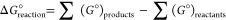
7where Δ*G*_reaction_^0^ is the free energy change associated with the elementary reaction on the alloy and *G*_X_^0^ is the free energy of reactant/product X, including entropy at 298 K and ZPE. Nørskov’s computational hydrogen electrode (CHE) model^37^ was used for the deprotonation steps to compute the free energy of H^+^ + e^–^, as shown in eq 8.
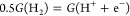
8

Applying a potential *U* to the electrode shifts the free energy of the potential-dependent reactions by |*e*|*U*, where |*e*| is the absolute charge of electrons.^37^

## 3 Results and Discussion

### 3.1 Characterization

#### 3.1.1 X-ray Diffraction

**Figure 1:**
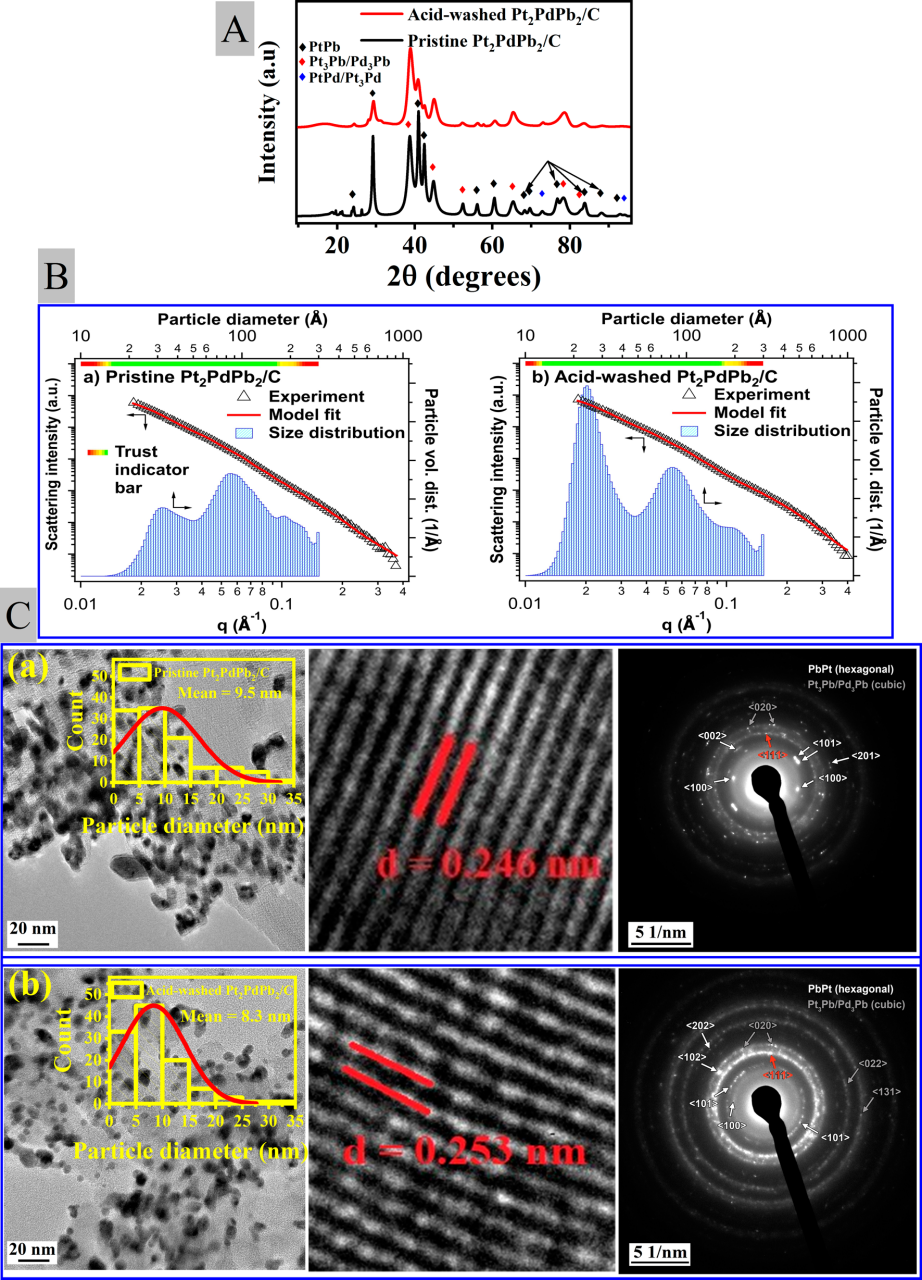
(A) XRD patterns of pristine and acid-washed (in 0.6 M HNO_3_ for 1 h) Pt_2_PdPb_2_/C. (a,b) of (B) are the SAXS profiles and the corresponding PSD of pristine and acid-washed Pt_2_PdPb_2_/C samples, respectively. (C) HR-TEM images at 20 nm resolution, *d*-spacing, and the indexed SAED of the Pt_2_PdPb_2_/C (a) pristine and (b) acid-washed Pt_2_PdPb_2_/C. Histogram of the PSD is shown in the insets depicted in images (a,b) of (C).

The wide-angle powder XRD patterns of the pristine and HNO_3_-washed Pt_2_PdPb_2_/C (at 0.6 M for 1 h—the optimum treatment condition that gave the best electrochemical results) are shown in Figure 1. The peaks were compared for the various phases in the XRD database. In the pristine Pt_2_PdPb_2_/C, the majority of the diffraction peaks showed the best match with cubic Pt_3_Pb/Pd_3_Pb (51%) (PDF 96-153-8800) and hexagonal PtPb (49%) (PDF 00-006-0374) (Figure 1a) with average crystallite sizes of 4.4 and 8.7 nm, respectively, determined by averaging the crystallite sizes calculated from each plane associated with these phases using Scherer’s equation. The presence of these phases was also evidenced by the comparison of the XRD patterns of Pt_2_PdPb_2_/C with those of the prepared Pt_3_Pb/C and PtPb/C binary metal catalysts (Figure S1). Because the Pt and Pd phases had similar crystalline diffraction patterns, it was difficult to assign the characteristic diffraction peaks observed at 2θ = 52, 73, 78, and 82° to either Pt_3_Pb or Pd_3_Pb. Hence, it was not possible to deduce from the XRD analysis whether Pb formed alloys with Pt or Pd in these phases. Minor diffraction peaks corresponding to PtPd/Pt_3_Pd are also observed at 2θ = 73 and 94°. The diffraction peaks corresponding to the Pt_3_Pb phase of pristine Pt_2_PdPb_2_/C showed a 0.5–1.5° negative shift relative to the binary catalyst Pt_3_Pb/C caused by the incorporation of a third metal, Pd, in our catalyst, indicating the presence of the ternary phases.^4,17^

By comparing the XRD patterns of Pt_2_PdPb_2_/C before and after acid treatment, we noticed that only the PtPb phase was strongly altered by the acid-washing step. This was confirmed by the complete disappearance (indicated by the arrows) or a partial decrease in the intensity of the XRD peaks corresponding to the PtPb phase (Figures 1 and S1). However, the PtPb phases were still observed in the acid-washed Pt_2_PdPb_2_/C catalyst. In contrast, Pt_3_Pb phase peaks in Pt_2_PdPb_2_/C remained unchanged (Figures 1 and S1). This is attributed to the PtPb phase component being thermodynamically less stable.^38^ ICP analysis of the solid product after acid treatment in 0.6 M HNO_3_ for 1 h (optimum treatment condition for best electrochemical results) revealed only Pb removal from the PtPb phase of Pt_2_PdPb_2_/C catalysts without affecting the Pt (Table S2). Hence, one could expect the appearance of new peaks corresponding to Pt nanoparticles or other phases formed because of this Pt/Pb compositional change. Nonetheless, the XRD pattern of the acid-washed Pt_2_PdPb_2_/C did not show any new diffraction peaks. Therefore, it is reasonable to conclude that acid-washed Pt_2_PdPb_2_/C possesses Pt, PtPb, and Pt_3_Pb/Pd_3_Pb phases. To better understand this leaching process, the XRD patterns of the pristine and acid-washed PtPb/C catalysts were also recorded and are displayed in Figure S1. Post acid washing, new peaks that matched elemental Pt were formed. However, these new peaks are less sharp than those of the Pt and PtPb phases (at 2θ = 24, 29, and 49°), suggesting that both Pt and PtPb remained available after the acid washing of PtPb/C, at least in the particle bulk.

The exact amount of each metal removed by the acid treatment was determined as follows: a Pt_2_PdPb_2_/C-coated Toray paper coated with a Pt/Pd/Pb concentration ratio of 3:0.8:3.2 ppm was immersed in solutions of varying HNO_3_ concentrations for 1 h, followed by washing with aqua regia to recover the metals that remained on the electrode. The ICP spectra of both solutions were measured after suitable dilution (0.5–5 ppm). As shown in Table S2, dipping the electrode in the HNO_3_ solutions primarily dissolved the Pb component of the nanoparticles and the Pd and Pt components to a much lesser extent. In addition, the amount of Pb removed from the sample increased with an increase in the HNO_3_ concentration (7% at 0.3 M to nearly 50% at 1.2 M). No measurable loss of Pt and Pd was detected, even at high concentrations used in the acid treatment step. This was further confirmed by the XRD showing peaks that matched elemental Pt only in the acid-washed PtPb/C control sample and not in the acid-washed Pt_3_Pb/C control sample.

#### 3.1.2 Small-Angle X-ray Scattering

The SAXS analysis of pristine and acid-washed Pt_2_PdPb_2_/C exhibited a roughly bimodal PSD ranging from 1 to 30 nm (Figure 1B, parts a and b) for both samples. The obtained PSD for the acid-washed sample clearly showed a shift to smaller diameters (Figure 1B, part b) compared to the PSD of the pristine sample (Figure 1B, a). The average diameter calculated from the displayed PSDs dropped by 14% from 14.6 nm (pristine sample) to 12.5 nm (acid-washed sample). The average particle sizes were significantly larger than those obtained using HR-TEM. Presumably, this difference was mainly due to the approximation used in modeling the SAXS data, where only the distribution of compact noninterfering spheroid particles was taken into account, disregarding the aggregation of primary nanoparticles. Nonetheless, the decrease in the average particle size after acid-washing is clearly proven by SAXS, and the proportional decrease in the average particle size was virtually the same as that indicated by HR-TEM.

#### 3.1.3 High-Resolution Transmission Electron Microscopy

The HR-TEM images of the Pt_2_PdPb_2_/C catalyst ink dropped on a TEM grid before and after acid treatment of the powder by mild stirring in 0.6 M HNO_3_ for 1 h are shown in Figure 1C, parts a and b. It can be seen that metal nanoparticles were well dispersed on the carbon support with few local agglomerations mainly in the case of pristine Pt_2_PdPb_2_/C. A clear improvement in dispersion was observed after the acid treatment process. After acid washing, the STEM-EDS mapping of the elements in the catalyst (Figure S2) showed a mapping color change only in the case of elemental Pb, indicating that HNO_3_ treatment caused an alteration in the Pb content. This is also evident from the reduction in the atomic percentage of Pb from 21 to 7% while showing no significant difference for Pt and Pd (Figure S2a,b). However, the changes in the atomic ratios measured by STEM-EDS mapping were slightly different from those measured by ICP, possibly because of localized measurements in the case of STEM-EDS mapping.

The HR-TEM images of the catalyst before and after acid treatment were analyzed using ImageJ software, and the extracted diameter distribution of the nanoparticles is presented in the inset histograms in Figure 1C, parts a and b, respectively. The PSD calculated by taking more than 100 randomly selected nanoparticles from the HR-TEM images showed a trend similar to that observed by SAXS. The average particle diameter reduced from 9.5 to 8.3 nm after the 0.6 M HNO_3_ treatment for 1 h. This significant decrease is attributed to the dissolution of the Pb component of the catalyst, which has a larger atomic size than those of Pt and Pd. However, it also suggests that the selective removal of Pb occurs not only from the surface but also from the bulk of the particles. However, the HR-TEM-measured average particle diameters are slightly different from the values obtained from SAXS, likely owing to the interference of the scattered waves by the closely packed particles during powder SAXS measurements and the spherical particle model applied while simulating the SAXS data. HR-TEM clearly shows that even though the majority of the particles were spherical, elliptical particles in addition to other shapes were present in the sample.

Electron diffraction analysis was performed on the HR-TEM images to analyze the crystal structure of Pt_2_PdPb_2_/C before and after acid treatment.^39^ The indexed SAED pattern is shown in Figure 1C, parts a and b. Average interplanar spacing of 0.246 and 0.253 nm were determined for the pristine and acid-washed samples, respectively. These values were assigned to the (111) facet of the Pt_3_Pb cubic crystal structure. The corresponding *d*-spacings for the (111) facets of the pristine and acid-washed catalysts, as measured by XRD, were 0.235 and 0.243 nm, respectively, confirming the similar effects of acid washing by both methods. This slight increase in the *d*-spacing after acid washing determined by HR-TEM could be due to the lattice space defects that remained after Pb leaching.^40^ Similar to the XRD observations, the processed SAED pattern of both samples displayed in Figure S4 also confirmed that the peaks corresponding to the relatively less stable PtPb phase were strongly affected by the acid treatment step as compared with those of the Pt_3_Pb phase in the sample.

#### 3.1.4 STEM-Energy Dispersive X-ray Spectroscopy

**Figure 2:**
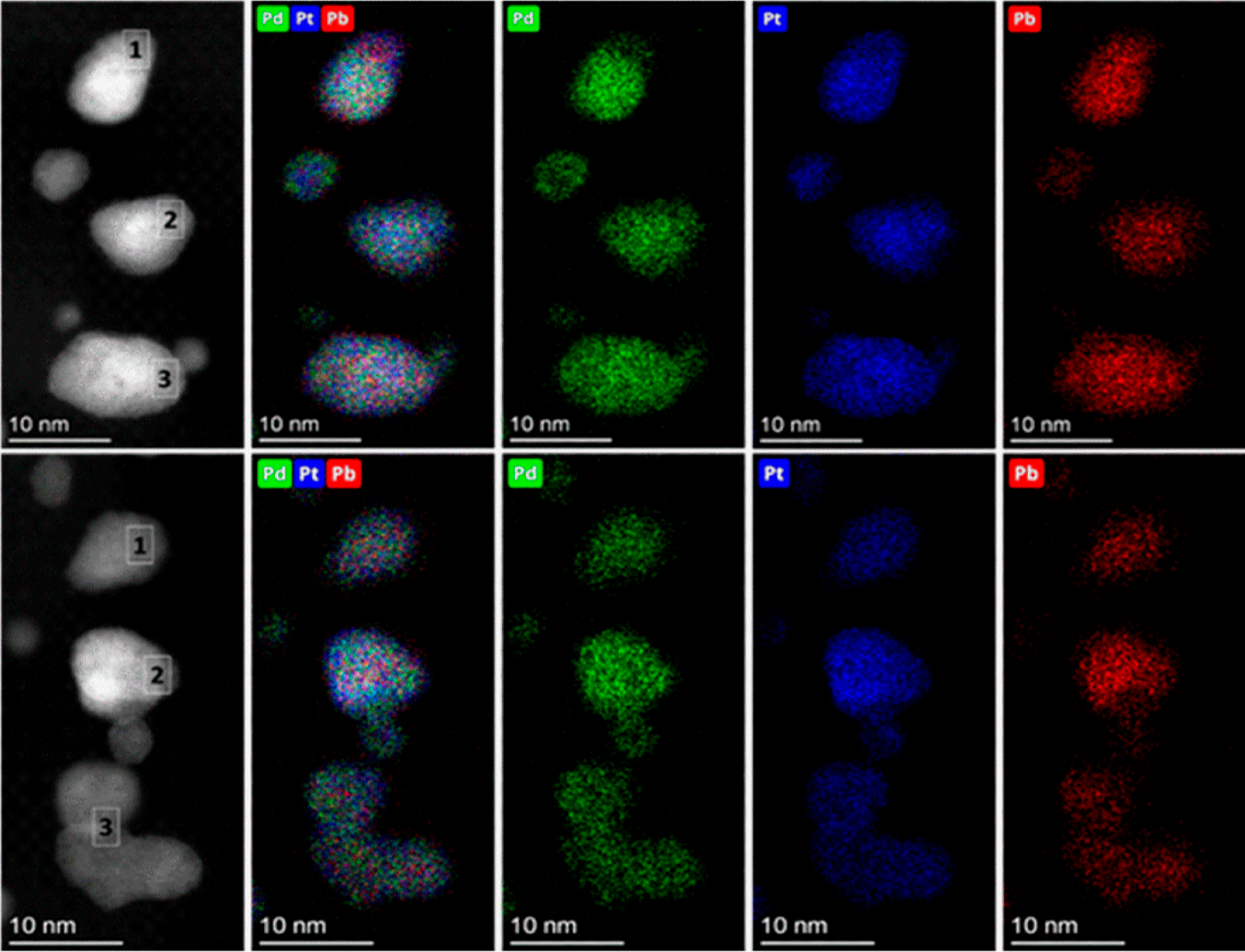
Typical STEM HAADF micrographs (left) and EDS maps of selected elements in pristine (top row) and acid-washed (bottom row) samples.

The signals from the major edges of Pt, Pd, and Pb in the sample were extremely weak, making electron energy loss spectroscopy (EELS) measurements unfeasible for Pt and Pb. The major edges are located above the 2000 eV level where substantial probe current would be needed which will lead to irreversible beam damage of the probed material during the data acquisition. Nevertheless, we have made efforts to provide relevant STEM-EDS maps with nanometric resolution (Figures 2 and S3), along with a compositional table (Tables 1 and S3). These data prove that there was a loss of Pb after the acid washing process, which correlates well with the findings from the XPS, ICP, and SEM-EDS measurements of the bulk analysis.

**Table 1:** EDS Compositional Results for Marked Individual Particles in Figure 2 and Typical Averaged Value (at Least 10 Particle Groups)

pristine		particle 1		particle 2		particle 3		average
element	family	at. %	at. % error	at. %	at. % error	at. %	at. % error	at. %
Pd	L	24.02	1.76	23.64	1.79	23.66	1.75	23.8
Pt	L	53.31	3.03	57.74	2.93	54.52	3.01	54.8
Pb	L	22.67	2.33	18.62	2.03	21.82	2.27	21.4

acid-washed		particle 1		particle 2		particle 3		average
element	family	at. %	at. % error	at. %	at. % error	at. %	at. % error	at. %
Pd	L	20.23	1.63	19.11	1.57	21.32	1.69	24.4
Pt	L	61.18	2.89	64.26	2.78	61.52	2.85	57.8
Pb	L	18.59	2.07	16.62	1.91	17.16	1.93	17.8

#### 3.1.5 X-ray Photoelectron Spectroscopy

**Figure 3:**
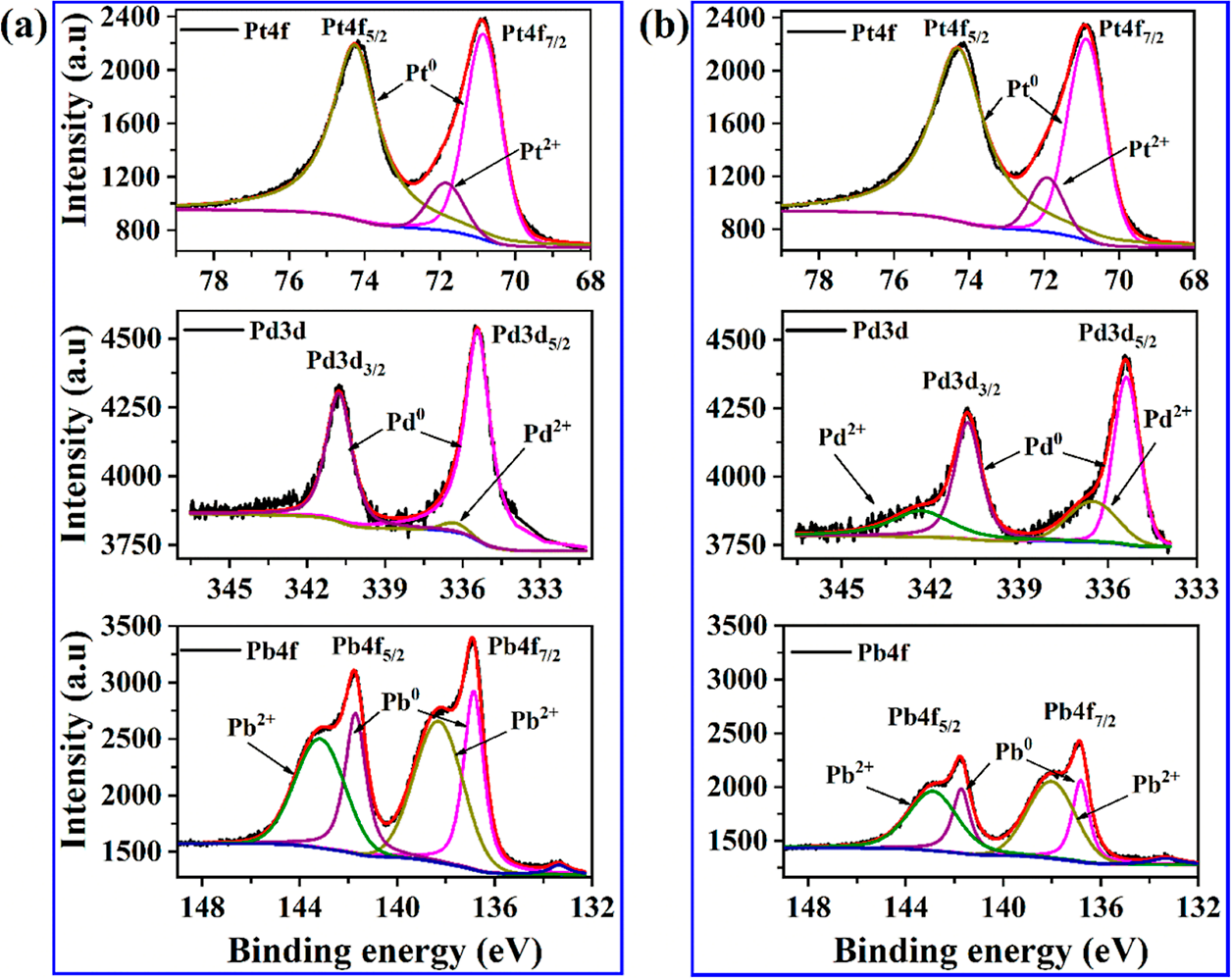
XPS spectra of Pt_2_PdPb_2_/C (a) before and (b) after acid treatment of the catalyst.

The XPS spectra of the metal constituents in Pt_2_PdPb_2_/C before and after acid treatment of the catalyst are displayed in Figure 3a,b, respectively, plotted on the same intensity scale to clearly visualize the change. The binding energy (BE) of C 1s (284.6 eV) was utilized as an internal standard for the recorded BE of Pt 4f, Pd 3d, and Pb 4f. The integrated areas of the deconvoluted XPS peaks of the metals in different oxidation states are summarized in Table S4. In both samples, two peaks of Pt 4f were observed at 71.9 and 74.3 eV corresponding to Pt 4f_7/2_ and Pt 4f_5/2_, respectively. The deconvoluted peak areas of Pt 4f of the Pt_2_PdPb_2_/C show that the majority (92%) of the Pt exists in a metallic state (Pt^0^) (peaks at 71.9 and 74.3 eV), while the remaining Pt is present as Pt^2+^ (peak at 71.8 eV). Acid washing did not alter either the content or oxidation state of Pt, considering the peak areas of the overall and specific oxidation states.

Likewise, the XPS spectra for Pd 3d showed two peaks at 335.4 and 340.8 eV related to Pd 3d_5/2_ and Pd 3d_3/2_, respectively, which were assigned to metallic Pd (Pd^0^). From the deconvoluted peak areas of Pd 3d of the pristine Pt_2_PdPb_2_/C, palladium is mostly (98%) present in a metallic state with minor Pd^2+^ at 336.3 eV. Similar to Pt, the treatment did not change the total Pd content on the surface because the overall deconvoluted areas remained unchanged. This also agrees with our elemental analysis using STEM-EDS and ICP. However, the acid washing procedure increased the oxidized state of Pd (Pd^2+^) to 36%. After acid treatment, the areas of spectra related to the metallic Pd have been reduced, a new peak that corresponds to Pd^2+^ was formed at 343 eV, and the area of the Pd^2+^ peak at 336.3 eV increased significantly, suggesting the oxidation of Pd.

The XPS spectrum for the Pb species in pristine Pt_2_PdPb_2_/C shows Pb 4f_7/2_ and Pb 4f_5/2_ at 136.9 and 141.8 eV, respectively, corresponding to the metallic state of Pb (Pb^0^). Deconvolution of the Pb spectrum provided two additional peaks of Pb^2+^ at 138 and 143 eV.^41^ In the pristine Pt_2_PdPb_2_/C, unlike Pt and Pd, the majority of Pb was present in the oxidized state (59%). It is clear from Figure 3 that post acid treatment of the catalyst an overall decrease in the area of the spectrum for Pt^0^ and Pb^2+^ was observed signifying the removal of Pb species. This result is consistent with the decrease in the Pb content measured by ICP and STEM-EDS. The formation of PbO_2_ species after acid washing was evidenced by an increase in the Pb^2+^/Pb^0^ ratio (56 to 72%). We believe that the removal of Pb from the surface exposes more active metals (Pt and Pd) for DME oxidation. XPS spectra of C 1s before and after acid washing of the catalyst were compared (Figure S5). No noticeable change was observed in the area of the spectra corresponding to the C–O bond at 285.76 eV (Table S4), indicating that the carbon support was not oxidized following acid washing.

### 3.2 Electrochemical Measurements

#### 3.2.1 Activity Comparison of PtPdPb-Based Catalysts

**Figure 4:**
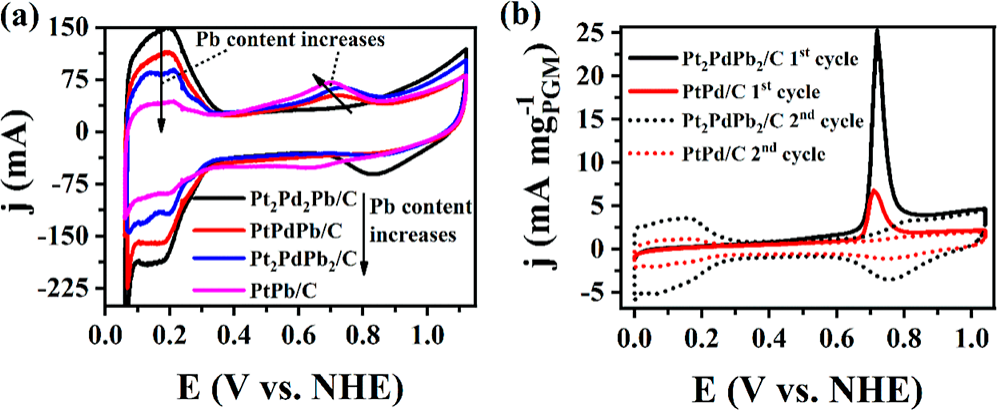
(a) CV recorded in N_2_-saturated 0.5 M H_2_SO_4_ for Pt_*x*_Pd_*y*_Pb_*z*_/C catalysts with varying Pb content (100 mV s^–1^). (b) CO_ads_ oxidation using PtPd/C and PdPdPb/C. (0.5 M H_2_SO_4_ electrolyte, 50 μg cm^–2^ catalyst loading, scan rate 10 mV s^–1^).

Pt_*x*_Pd_*y*_Pb_*z*_/C-based catalysts with various metal atomic ratios were synthesized. ICP analysis confirmed that the atomic ratios were nearly equal to the expected nominal values (Table S5). The electrochemical behavior of these catalysts was tested in a three-electrode cell in a N_2_- and DME-saturated 0.5 M H_2_SO_4_ solution. Figure 4a shows the cyclic voltammetry (CV) curves recorded in a N_2_-saturated solution at 100 mV s^–1^ for samples with varying Pb content. As the Pb content increased, the anodic and cathodic currents of the hydrogen underpotential desorption region (*H*_UPD_) (0.05–0.35 V) decreased, indicating that the electrochemical hydrogen adsorption and desorption currents of the ternary metal particles were modified by changes in the Pb composition. Pd is well-known for its strong hydrogen adsorption capabilities, which explain the comparable *H*_UPD_ observed in the cyclic voltammetry (CV) recorded using Pt/C and PtPd/C catalysts (Figure S6). Consequently, the change in the hydrogen underpotential deposition (*H*_UPD_) is not associated with the coverage of Pt by Pd. Figure S7 shows the change in the charge at *H*_UPD_ versus the Pb content. The electrochemically active PGM sites were diluted to a higher Pb content. Moreover, a Pb-oxidation peak with a peak potential of 0.7 V was observed. Huang et al. observed that Pb was oxidized at a lower potential than that for Pt and Pd, whose oxidations start at 0.8 V.^42^ The Pb-oxidation peak current increased, and its onset potential decreased to a lower-potential region as the Pb content increased. Pb oxide formation at a lower potential (0.4–0.8 V) prohibits the oxidation of the active metals, Pt and Pd, which is confirmed by the suppression of the Pt/Pd oxide formation peaks beyond 0.8 V as Pb content increases. Consequently, this resulted in the flattening of the Pt/Pd reduction cathodic peak at 0.84 V in the reverse scan. The oxidation of Pt is essential to provide oxygen-containing species to facilitate the removal of surface-poisoning species, CO_ads_, via a bifunctional mechanism.^43^ Hence, the beneficial role of Pb, which is discussed in the following sections, was not realized in its high content as it dilutes and hinders the oxidation of the electrochemically more active metals.

CV measurements in a DME-saturated solution using the synthesized Pt_*x*_Pd_*y*_Pb_*z*_/C-based catalysts are presented in Figure S8. The catalyst with the lowest Pb content, Pt_2_Pd_2_Pb/C, showed the best performance in terms of DME oxidation peak current (*j*_p_) (14.58 mA mg_PGM_^–1^) and charge (*Q*_oxi_) 79.60 mC mg_PGM_^–1^ [calculated by integrating the DME oxidation region (0.6–1 V)]. Nonetheless, *j*_p_ and *Q*_oxi_ of the Pb-free binary metal catalyst, PtPd/C, (5.09 mA mg_PGM_^–1^ and 32.47 mC mg_PGM_^–1^, respectively) showed results lower than those for Pt_2_Pd_2_Pb/C, suggesting the ligand contribution and structure optimization of Pb alloying. Pb is more electronegative and has different lattice size parameters that alter the electronic properties and introduces strain into the crystal lattice of certain phases in nanoparticles. These changes impede poisoning of the primary PGM-active sites by intermediate CO_ads_.

To investigate the electronic effect of Pb on the CO tolerance of the catalyst, oxidative stripping of preadsorbed CO was performed using carbon-supported PtPd/C and PtPdPb/C catalysts. CO stripping measurements were performed according to a procedure described elsewhere.^44^ In short, a 0.5 M H_2_SO_4_ solution was saturated with CO, and the catalyst-coated glassy carbon working electrode was held in the solution at 0.05 V for 1 min to adsorb the CO on the surface. The excess CO present in the solution was removed by purging N_2_ into the solution, and the CV was measured at 10 mV s^–1^ in the potential range of adsorption and full oxidation of CO (0.05–1.1 V). Figure 4b compares the first two CV cycles recorded during the oxidation of the surface preadsorbed CO using the PtPd/C and PtPdPb/C catalysts. In both cases, the adsorbed CO was oxidized in the first cycle, and the subsequent cycle leveled off to the background level. PtPdPb/C exhibited a CO_ads_ oxidation onset potential that was nearly 50 mV lower than that of PtPd/C. This suggests that the addition of Pb altered the surface electronic structure of the nanoparticles, and the interaction contributed to the improved tolerance of the catalyst to CO_ads_. Various reports have shown that doped oxophilic metals facilitate C–O bond breaking.^45,46^ Moreover, the higher CO_ads_ oxidation charge (*Q*_co_) (88.67 ± 5.07 mC mg_PGM_^–1^) of PtPdPb/C than PtPd/C (16.03 ± 2.92 mC mg_PGM_^–1^) indicates the contribution of the Pb in improving the electrochemically active sites by increasing the Pt–Pt distance.^22^ Therefore, the change observed in the *H*_UPD_ region following the dealloying of Pb can be attributed to the alteration in the electronic effect induced by Pb removal.

To evaluate the CO adsorption characteristics of Pt/C, Pd/C, and Pb/C, we conducted CO stripping experiments and examined the individual features of these three metals (Figure S9). We observed a CO oxidation peak in the first cycle of CV using Pt/C and Pd/C catalysts. However, in the case of Pb/C, the first cycle overlapped with the consecutive cycles, resulting in the absence of a CO oxidation peak. Hence, it is reasonable to conclude that CO adsorption occurs to a significant extent on Pt and to varying extents on Pd, but not on Pb. By comparing the CO oxidation charge between Pt/C and Pd/C (5.93 × 10^–5^ vs 1.29 × 10^–5^ C), we calculated that the majority of CO was adsorbed on Pt, while the remaining portion was adsorbed on Pd.

#### 3.2.2 Effect of HNO_3_ Concentration and Treatment Time on Pt_2_PdPb_2_/C

We have seen from the various characterizations in the previous sections (XRD, ICP, STEM-EDS, and XPS) that acid treatment mainly reduced the Pb content of the catalyst. Complete removal of Pb, however, resulted in PtPd, which was shown to have little or no activity toward DME oxidation. Moreover, it is important to focus on the device-level objective of noble metal content reduction in the catalysts. Therefore, despite the higher DME oxidation activity of the Pt_*x*_Pd_*y*_Pb_*z*_/C catalyst with a low Pb loading, we investigated the catalyst with the highest initial Pb content, Pt_2_PdPb_2_/C. Furthermore, the catalyst was subjected to varying concentrations of HNO_3_ acid to optimize the Pb content in the sample that will lead to the attainment of optimal DME oxidation activity. The following discussion is limited to the chosen Pt_2_PdPb_2_/C composition (pristine) and acid-washed Pt_2_PdPb_2_/C catalyst samples.

**Figure 5:**
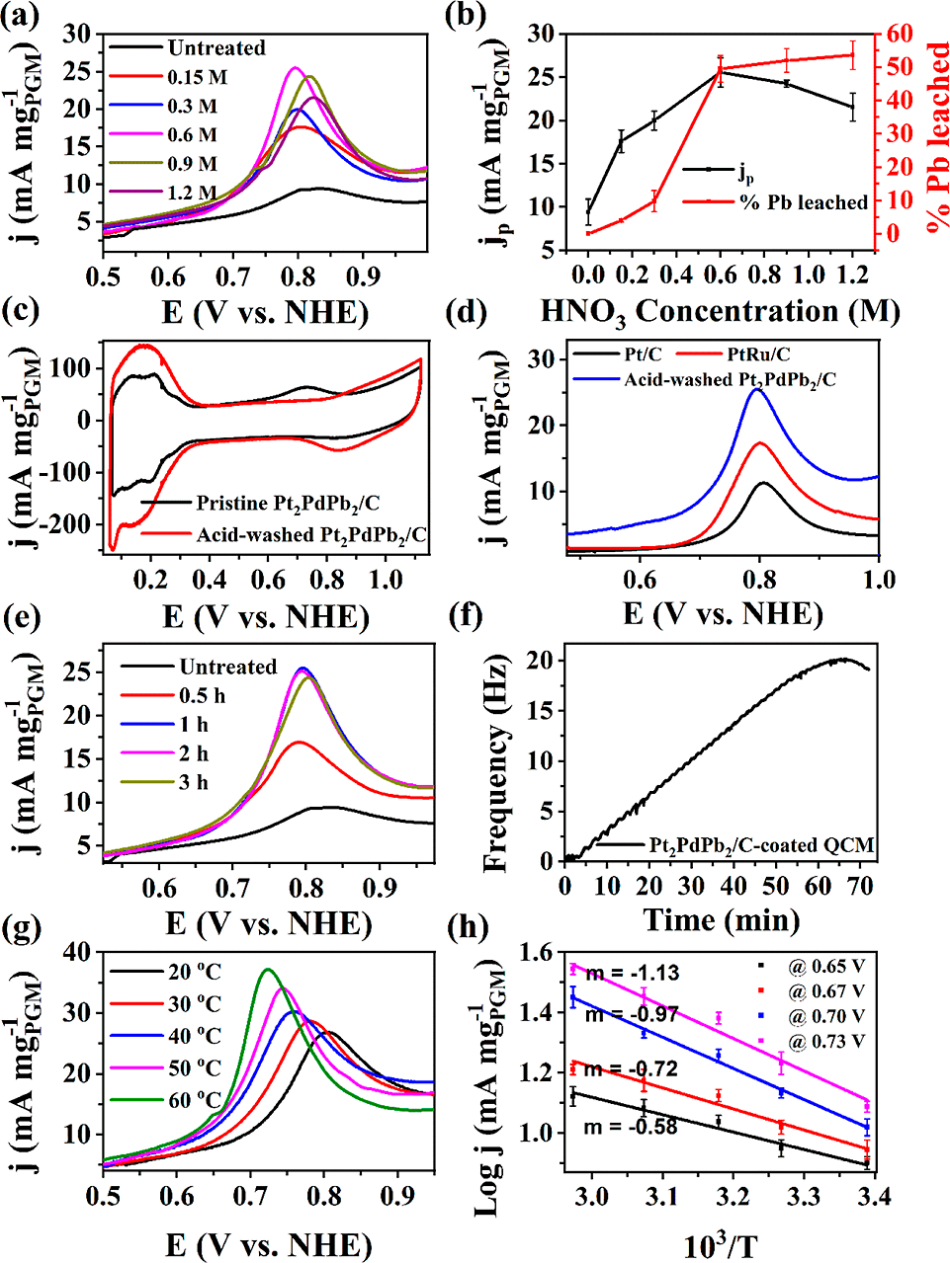
(a) Effect of HNO_3_ concentration treatment on the activity of Pt_2_PdPb_2_/C toward DME oxidation (b) % of Pb removed and *j*_p_ vs the HNO_3_ concentration used during treatment (c) CV in N_2_-saturated 0.5 M H_2_SO_4_ using pristine and acid-treated Pt_2_PdPb_2_/C, and (d) DME oxidation using acid-treated Pt_2_PdPb_2_/C, PtRu/C, and Pt/C. (e) Effect of Pt_2_PdPb_2_/C treatment time in 0.6 M of HNO_3_ on the activity of the catalyst for DME oxidation (f) mass change of Pt_2_PdPb_2_/C with time in 0.6 M of M HNO_3_ measured by QCM (catalyst loading ∼3 ng cm^–2^). (g) Effect of temperature on the DME oxidation. (h) Plot of the log *j* vs 1/*T*. (50 μg cm^–2^ catalyst loading, scan rate 10 mV s^–1^).

Figure 5c compares the CV recorded in a N_2_-saturated 0.5 M H_2_SO_4_ using the Pt_2_PdPb_2_/C-coated working electrode before and after dipping the electrode in HNO_3_ (the optimized HNO_3_ concentration and washing time of 0.6 M and 1 h, respectively). The electrochemical active surface area (ECSA), calculated from the total charge obtained by integrating the *H*_UPD_ region of Figure 5c,^44^ was increased from 16.07 ± 0.32 to 25.56 ± 0.66 m^2^ g_PGM_^–1^ after the treatment. This is because the removal of Pb from the surface exposed the active metals, as evidenced by the XPS spectra. Moreover, following the acid wash and removal of Pb, the Pb-oxidation peak at 0.7 V disappeared, and the Pt/Pd oxide formation region had been raised. Hence, the Pt/Pd oxide reduction peak was visible.

The activity of Pt_2_PdPb_2_/C, treated with HNO_3_ of varying concentrations, toward DME oxidation is displayed in Figure 5a (the forward scan in the region where DME oxidizes is shown for clarity). Table S6 summarizes the electrochemical parameters derived from these voltammograms. Highest *j*_p_ and *Q*_oxi_ of 25.66 ± 1.11 mA mg_PGM_^–1^ and 65.51 ± 1.46 mC mg_PGM_^–1^, respectively, were achieved following a 1 h immersion of the Pt_2_PdPb_2_/C-coated electrode in 0.6 M HNO_3_. These values were nearly 2- and 4-folds higher, respectively, than that obtained when the electrode was utilized untreated. The lower *j*_p_ and *Q*_oxi_ obtained with the Pt_2_PdPb_2_/C-coated electrode treated in a lower than 0.6 M HNO_3_ are ascribed to the inadequate removal of Pb from the catalyst’s outer layers, which could not overcome the negative role of high Pb content in the catalyst (Table S2). Moreover, *j*_p_ decreased and shifted to higher potentials when the electrode treatment was performed at HNO_3_ concentrations higher than 0.6 M (Figure 5a,b). Beyond 0.6 M HNO_3_, the more active metals, Pt and Pd, also start to dissolve in the acid in addition to Pb (Table S2).

The activity of the acid-washed Pt_2_PdPb_2_/C catalyst was also compared with that of commercial Pt/C and a state-of-the-art catalyst for DME electro-oxidation, PtRu/C (Figure 5d). The 0.6 M HNO_3_-treated Pt_2_PdPb_2_/C showed nearly 122 and 151% higher *j*_p_ (25.6 ± 1.1 vs 11.5 ± 0.8 mA mg_PGM_^–1^) and *Q*_oxi_ (65.5 ± 1.4 vs 27.2 ± 1.0 mC mg_PGM_^–1^), respectively, than those of the commercial Pt/C. The peak current density achieved with PtRu/C (17 ± 0.9 mA mg_PGM_^–1^) is significantly lower than that observed with the acid-washed Pt_2_PdPb_2_/C. As mentioned above, washing Pt_2_PdPb_2_/C for 1 h in 0.6 M HNO_3_ removed nearly 50% of the Pb content, leading to an identical composition of metals as that of the Pt_2_PdPb/C catalyst. However, the acid-washed Pt_2_PdPb_2_/C provided a higher ECSA, *j*_p_, and *Q*_oxi_ than the as-prepared Pt_2_PdPb/C (Figure S10b). Even though the acid-treated Pt_2_PdPb_2_/C and as-prepared Pt_2_PdPb/C had the same metal composition, their XRD patterns showed that the former catalyst contained additional peaks corresponding to the PtPb phase (Figure S11). Therefore, acid washing of Pt_2_PdPb_2_/C results in not only the removal of Pb but also causes structural changes in the catalyst by optimizing the PtPb phases in addition to the formation of some free Pt nanoparticles.

Because PtPb and Pt_3_Pb are the two dominant phases present in the Pt_2_PdPb_2_/C catalyst, a CV was recorded in N_2_- and DME-saturated electrolytes using pristine and acid-washed PtPb/C and Pt_3_Pb/C (Figure S12a,b) to further confirm which of these two phases were more affected by the acid treatment. A clear widening of the *H*_UPD_ region was observed after acid washing of PtPb/C than that in the case of Pt_3_Pb/C, indicating a significant removal of Pb from the PtPb/C and exposure of more active metal sites of the noble metals. This also shows that Pt_3_Pb is a thermodynamically metastable alloy that is less prone to dissolution in an aqueous acidic solution. The anodic peak of pristine PtPb/C seen at 0.7 V suggests that the loosely bound Pb in PtPb/C is in the oxide form, passivating and preventing the active elemental Pt from being oxidized. This is evident from the absence of a Pt reduction peak at 0.8 V in the reverse scan. However, this anodic peak was absent in the pristine and acid-washed Pt_3_Pb/C. Acid washing of Pt_2_PdPb_2_/C primarily led to a significant reduction in the surface coverage of the DME-inactive PtPb phases by removal of weakly alloyed Pb from the unstable PtPb phase. Extensive set characterization of the Pt_2_PdPb_2_ catalyst confirmed that the acid-washed Pt_2_PdPb_2_/C catalyst contained Pt, PtPb, and Pt_3_Pb phases. To determine which phases are involved in DME oxidation, we synthesized these phases separately and tested their electrochemical activity toward the DME oxidation reaction (Figure S10c–e). PtPb/C showed no significant difference with or without DME, but Pt_3_Pb/C and Pt/C displayed DME oxidation activity with peak current densities of approximately 13 and 7 mA mg_PGM_^–1^ (background subtracted current values), respectively, in a DME-saturated solution, indicating that these phases synergistically contribute to the electrochemical oxidation of DME.

Here, we report a 1 h HNO_3_ treatment of Pt_2_PdPb_2_/C as no significant improvement in the catalyst activity was observed after 1 h of exposure (Figure 5e). This is because, beyond 1 h of treatment in 0.6 M HNO_3_, no more Pb was removed from the catalyst, as verified by the change in mass vs acid treatment time measurement conducted using Pt_2_PdPb_2_/C-coated QCM (Figure 5f). The frequency increased by 20 Hz for up to 61 min, confirming a reciprocal decrease in the mass of the catalyst due to dissolution. Of the 600 ng of catalyst loaded on the 0.196 cm^2^ QCM electrode, close to 110 ng was dissolved in the HNO_3_ solution at the end of 61 min. Because the Pt and Pd compositions did not show a significant difference before and after the 1 h HNO_3_ treatment (Table S2), this decrease in the mass is ascribed to the dissolution of Pb. Accordingly, approximately 58% of the initial amount of Pb present in the catalyst has been dissolved. This is not significantly higher than the amount measured by the ICP after 1 h of treatment in 0.6 M HNO_3_ (∼50%). The slight increase in mass beyond 65 min could be due to the deposition of previously dissolved Pb.

#### 3.2.3 Effect of DME Oxidation Temperature

Figure 5g shows the dependence of the Pt_2_PdPb_2_/C activity on the DME oxidation temperature. The measurements were performed between 20 and 60 °C, and the electrochemical parameters are summarized in Table 2. It should be noted from the data points that with an increase in temperature, the DME oxidation current increased. At 60 °C, the peak current density and oxidation charges were 7.5 mA mg_PGM_^–1^ and 86.8 ± 0.9 mC mg_PGM_^–1^, respectively, which are 50 and 30% increases relative to the values measured at 20 °C. Moreover, the onset and peak potentials at 60 °C are shifted to lower potential regions by 35 and 75 mV, respectively, as compared to those at 20 °C. The DME oxidation temperature improves the activity of the catalyst by facilitating the removal of surface-poisoning species such as CO_ads_.^10^ At higher temperatures, the oxidation and removal of these species were improved. Moreover, water activation, which results in −OH_ads_ formation, is facilitated at high temperatures and enhances DME oxidation.

**Table 2:** Summary of Anodic Parameters of Pt_2_PdPb_2_/C in DME Oxidation at Selected Temperatures

temperature (°C)	*j*_p_ (mA mg_PGM_^–^^1^)	*Q*_oxi_ (mC mg_PGM_^–^^1^)	onset potential (V vs NHE)	peak potential (V vs NHE)
20	5.10 ± 0.14	67.36 ± 0.98	0.54 ± 0.02	0.81 ± 0.00
30	5.67 ± 0.00	75.00 ± 0.62	0.53 ± 0.04	0.78 ± 0.01
40	5.83 ± 0.23	78.47 ± 1.08	0.52 ± 0.00	0.76 ± 0.01
50	6.83 ± 0.16	81.94 ± 1.96	0.51 ± 0.00	0.74 ± 0.00
60	7.50 ± 0.00	86.81 ± 0.98	0.51 ± 0.02	0.73 ± 0.01

To understand the kinetics of DME oxidation on acid-washed Pt_2_PdPb_2_/C, the activation energy (*E*_a_) was determined using Arrhenius eq 9.
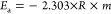
9where *R* is the universal gas constant and *m* is the slope of the Arrhenius plot obtained by plotting log *j* vs 1/*T* (Figure 5h). The *E*_a_ calculated from the direct linear relationship of log *j* vs 1/*T* over the tested temperature region ranges from 11 kJ mol^–1^ at 0.65 V to 21 kJ mol^–1^ at 0.73 V. Outside of this potential region, with an increase in the oxidation temperature, the log *j* vs 1/*T* starts to be inversely related due to the negative shift of the onset and peak potentials, as shown in Table 2. This *E*_a_ value is lower than the previously reported *E*_a_ values of DME oxidation on binary [PtRu/C (57 kJ mol^–1^) and Pt_0.75_Pd_0.25_/SnO_2_/C (46.5 kJ mol^–1^)] and ternary [Pt_3_Pd_3_Sn_2_/C (48.7 kJ mol^–1^)] metal catalysts, indicating more facile DME oxidation on acid-treated Pt_2_PdPb_2_/C.^10,17,47^

#### 3.2.4 Constant Potential Stability Test

**Figure 6:**
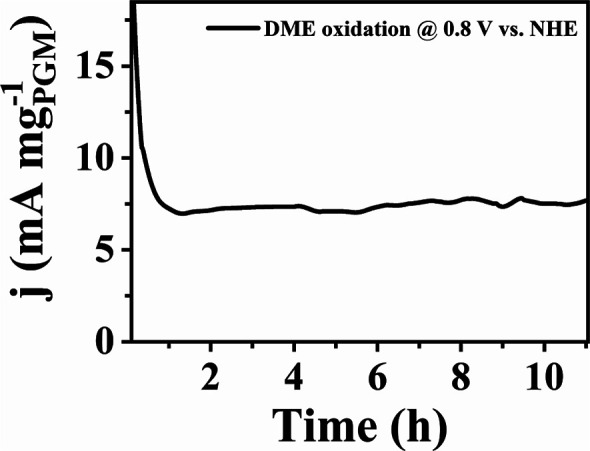
Current measured at 0.8 V vs NHE using acid-washed Pt_2_PdPb_2_/C in an electrolyte saturated with DME (0.74 M) (catalyst loading 50 μg cm^–2^; electrolyte 0.5 M H_2_SO_4_).

A chronoamperometry experiment was conducted for 11 h at 0.8 V in a 0.5 M H_2_SO_4_ electrolyte saturated with DME (0.74 M) to assess the stability of the catalyst with maximum activity (refer to Figure 6). The applied potential of 0.8 V was chosen to be close to the DME oxidation peak potential (refer to Figure 5a), ensuring an accelerated durability test and that the stability study was conducted during the complete oxidation of DME. Despite an initial rapid decay in current density within the first hour (from 18 to 7 mA mg^–1^ PGM), the catalysts displayed a stable steady-state current beyond 1 h (∼7.5 mA mg^–1^ PGM at the end of the 11 h oxidation period). This indicates that the catalyst efficiently removed surface-adsorbed poisoning intermediates, such as −CO_ads_, −CHO_ads_, and –CH_3ads_, contributing to its stability.

An extended chronoamperometry experiment was conducted for 11 h using acid-washed Pt_2_PdPb_2_/C in solutions containing equal concentrations (0.74 M) of methanol and DME (Figure S13). The initial currents observed for methanol were higher than those observed for DME (35 vs 18 mA mg_PGM_^–1^), and this trend persisted for the first 3 h. However, while the current obtained from methanol oxidation gradually declined over time, that for DME oxidation remained stable. At the end of the 11 h oxidation period, DME exhibited a higher current than methanol (7.5 vs 4.3 mA mg_PGM_^–1^). It should be noted that the current and power outputs of DME-gas-fed fuel cells are significantly higher than those of fuel cells running on a liquid solution of methanol.^14^

### 3.3 Fuel Cell Measurement

**Figure 7:**
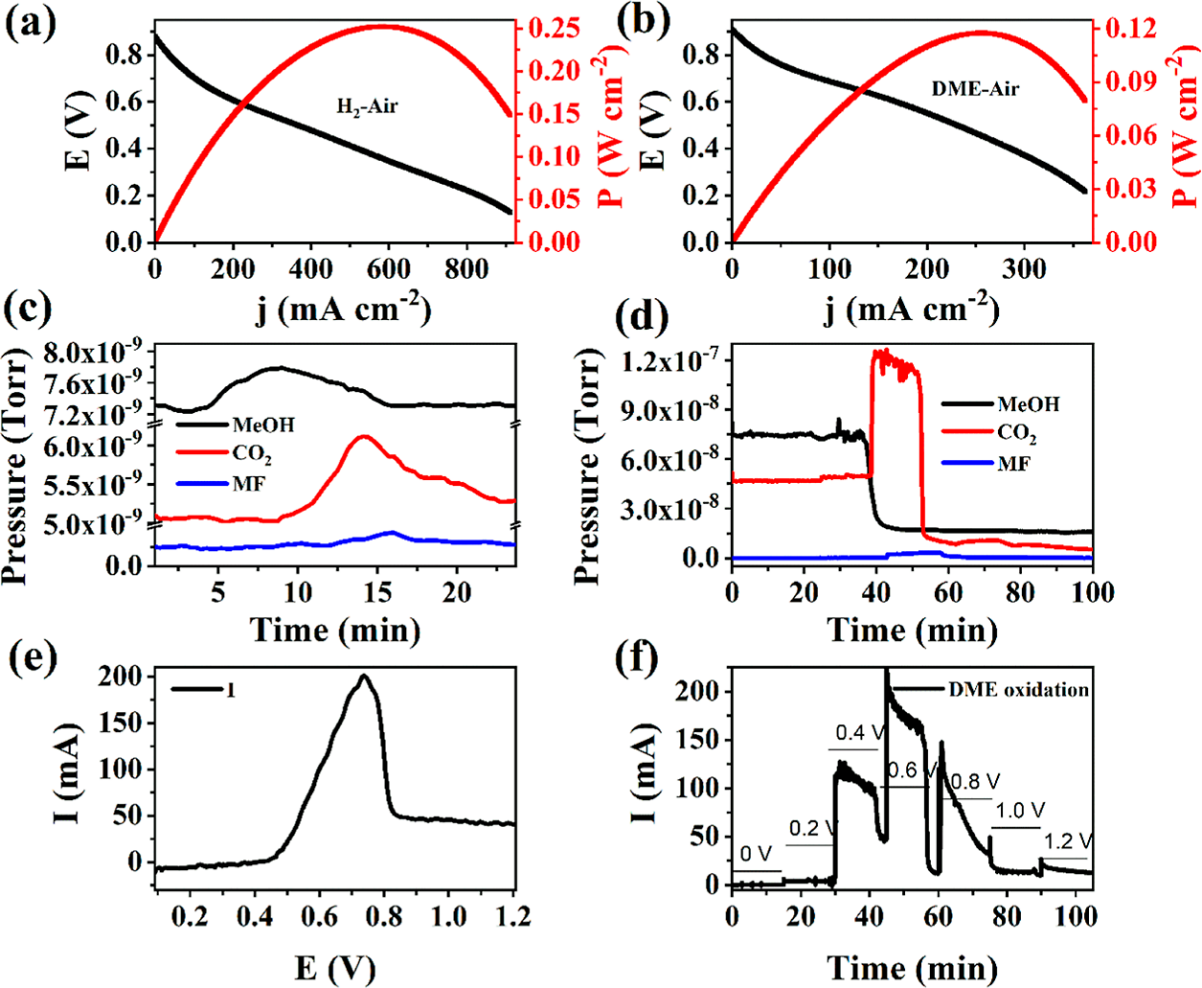
Polarization curve of the PEMFC operated with (a) H_2_–air (anode–cathode) and (b) DME–air using 1 mg_PGM_ cm^–2^ acid-treated Pt_2_PdPb_2_/C and 2.7 mg_PGM_ cm^–2^ Pt/C as anode and cathode catalysts, respectively (anode and cathode flow rate 40 and 350 mL min^–1^, respectively; cell temperature 70 °C). (c,d) Change in the mass signals of DME oxidation intermediates (MeOH, CO_2_, and MF) recorded during (e) cyclic voltammetry at 2 mV s^–1^ and (f) potential steps, respectively.

The polarization (*j*–*V*) curves of a PEMFC operated at 70 °C with H_2__air or DME_air (anode_cathode) using HNO_3_-treated Pt_2_PdPb_2_/C and Pt/C as anode and cathode catalysts are displayed in Figure 7a,b, respectively. The cell was initially operated with H_2_–air to evaluate the quality of the assembled cell and to use the results as a reference for the DDMEFC. The mass-normalized peak power densities for H_2_–air- and DME–air-operated fuel cells were 250 and 118 mW mg_PGM_^–1^ at 0.40 and 0.45 V, respectively, measured under a low cell temperature (70 °C), no backpressure (0 bar), and low anode catalyst loading (1 mg_PGM_ cm^–2^). A comparison of the DDMEFC power densities obtained in this study to previously reported values is presented in Table 3. The acid-treated ternary metal alloy anode catalyst composed of Pt, Pd, and Pb provided one of the highest power densities reported for a DDMEFC.

**Table 3:** Comparison of DDMEFC Performance of Different Catalysts Reported in the Literature

catalyst	Pt/C	Pt_0.8_Ru_0.2_/C	Pt_46_Ru_44_Pd_10_/C	SnO_2_/Pt/C	Pt_0.75_Pd_0.25_/SnO_2_/C	HNO_3_-treated Pt_2_PdPb_2_/C
anode loading (mg_Pt_ cm^–^^2^)	2	2	2.7	1.23	1.2	1
cathode loading (mg_Pt_ cm^–^^2^)	2	2	2	NA	3.5	2.7
air/O_2_ flow rate (mL min^–^^1^)	300	350	500	300	400	350
DME flow rate (mL min^–^^1^)	25	40	40	15	40	40
cell temperature (°C)	90	110	80	70	70	70
backpressure (bar)	2	3	1.4	0	0	0
power density (mW cm^–^^2^)	30	60	120	105	110	118
power density (mW mg_PGM_^–^^1^)	15	30	33	85	90	118
references	(49)	(8)	(50)	(51)	(10)	this study

The DDMEFC showed approximately 50 mV higher *E*_OC_ than that of the H_2_–air fuel cell (930 vs 880 mV). The transport of fuel from the anode to the cathode side of a fuel cell results in mixed potentials at the cathode, thereby lowering the overall cell voltage. Kashyap et al. measured crossover current densities of H_2_- and DME-operated fuel cells as 20 and 1.5 mA cm^–2^, respectively.^10^ DME is a larger molecule and has a higher dipole moment than that of H_2_, enabling it to have a lower crossover, and hence, a higher *E*_OC_. In this work, the crossover current was measured by applying a sufficiently high oxidation potential of 0.8 V vs *E*_OC_ on the cathode while flowing a 100% humidified DME at a flow rate of 40 mL min^–1^ through the anode and humidified N_2_ at a low flow rate of 10 mL min^–1^ through the cathode. It is important to note that humidified N_2_ was allowed at a low flow rate in the cathode to prevent Nafion membrane drying and to ensure sufficient residence time for the fuel that may crossover from the anode to the cathode. A steady-state crossover current of 7 mA cm^–2^ was observed for the direct DME fuel cell (Figure S14). The quantification of DME crossover involved approximating the measured crossover current (*I*_cross_) as the permeation rate (*n*_perm_) using Faraday’s law as described by eq 10. Consequently, the calculated permeation rate for the DME fuel cell was determined to be 8.71 × 10^–7^ mol cm^–2^ s^–1^, which is lower than the methanol permeation rates (5.53 × 10^–7^ mol cm^–2^ s^–1^) reported in previous studies employing an even thicker membrane (160 μm) than that used in our study (50 μm).^48^
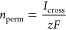
10where *z* and *F* represent the number of electrons transferred and Faraday’s constant (96,385.33 C mol^–1^), respectively. Given the high potential (0.8 V) applied to oxidize the crossed fuel, complete oxidation was assumed, resulting in a value of *z* of 12 electrons.

### 3.4 Online Mass Spectroscopy

Figure 7c and d shows the change in the mass signals of the intermediates MeOH, CO_2_, and methyl formate (MF) detected during DME oxidation on acid-treated Pt_2_PdPb_2_/C potentiodynamically (Figure 7e) and potentiostatically (Figure 7f). The mass signals of MeOH, CO_2_, and MF were detected at mass-to-charge ratios (*m*/*z*) of 31, 44, and 60, respectively. Formic acid, a widely reported DME oxidation intermediate, could not be detected because of the overlap of its mass signals with those of DME at all possible *m*/*z* ratios (29, 45, and 46). No DME oxidation current was observed; hence, no mass signal for the intermediates relative to the background was observed up to 0.2 V. The small current observed starting at 0.2 V was due to the formation of methanol, which was also evident by the increase in its mass signal at this potential. The methanol mass signal peaked at ∼0.45 V. The mass signals of CO_2_ started to sharply increase at 0.45 V in connection with the methanol signal decrease, suggesting that the formation of methanol precedes CO_2_ formation, and methanol oxidizes to CO_2_. This is in agreement with the previously reported results.^10^ The maximum mass signal of the CO_2_ coincides with the corresponding Faradaic peak current of DME oxidation at 0.73 V. Beyond this potential, the mass signal decays gradually to the background level. The mass signal of MF appears within the time and potential range of the appearance of CO_2_, indicating that the formation of both intermediates occurs in parallel. A similar trend in the relationship between the mass signal and current of these intermediates was observed for constant potential application.

**Scheme 1:**
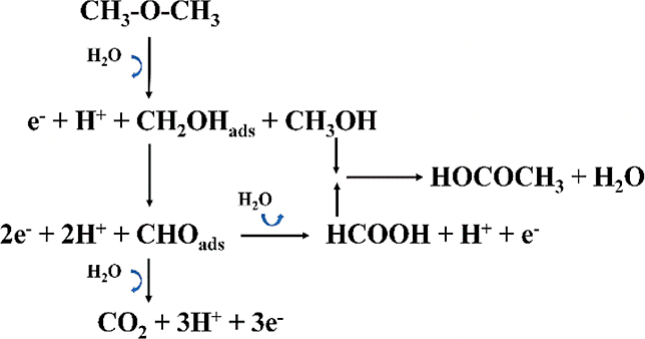
Proposed DME Oxidation Pathway

Based on the above observations, a possible DME oxidation mechanism is proposed as shown in Scheme 1. Initially (<0.45 V), DME adsorbed on the catalyst undergoes dissociation to form MeOH and CH_2_OH_ads_ via a one-electron pathway or dehydrogenates, at lower potentials, to CH_2_OH_ads_ via a two-electron pathway.

Following this, further dehydrogenation of CH_2_OH_ads_ (to CHO_ads_) and water activation yield either CO_2_ in a three-electron path or formic acid in a one-electron path. The formation of MF occurs in parallel with the evolution of CO_2_ from the reaction between methanol and formic acid or oxidation of the surface-adsorbed CH_2_OH_ads_.

### 3.5 DFT Calculations

**Figure 8:**
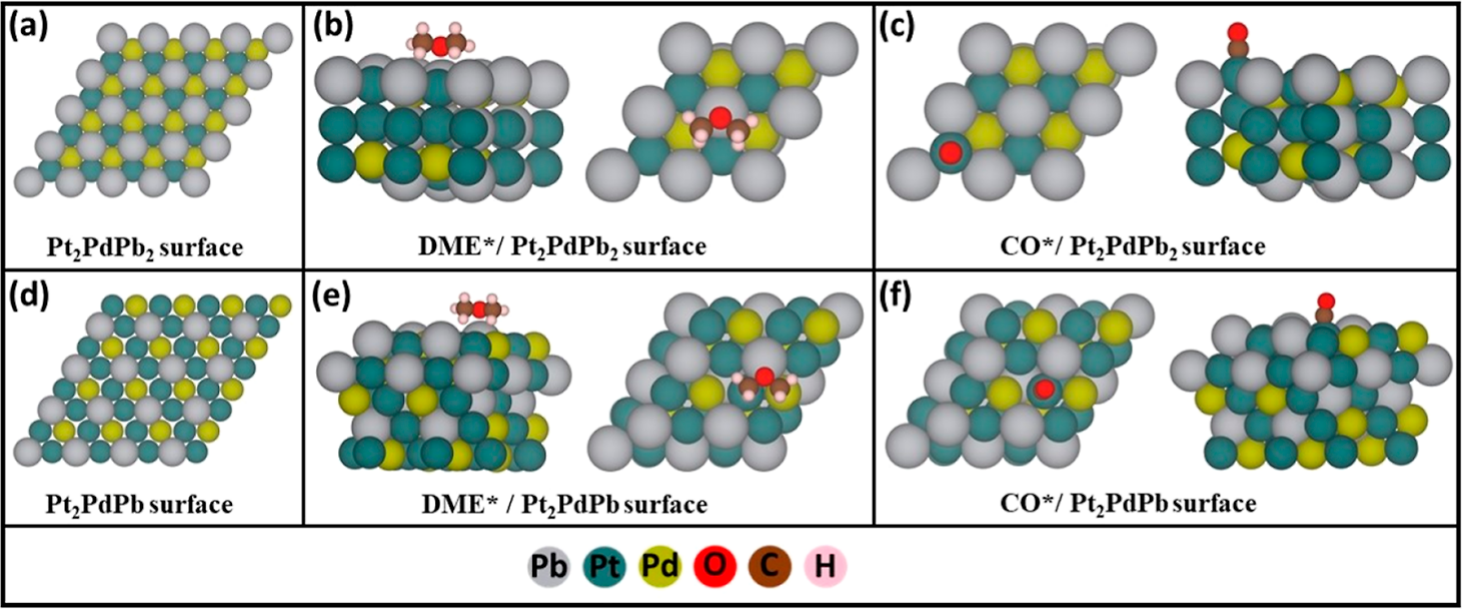
(a) Top view of the surface of Pt_2_PdPb_2_. (b) Most stable adsorption configuration of DME (top and side views) on Pt_2_PdPb_2_. (c) CO adsorption on top of Pt (top and side view) for Pt_2_PdPb_2_ surfaces. (d) Top view of the surface of Pt_2_PdPb. (e) Most stable adsorption configuration of DME (top and side view) on Pt_2_PdPb. (f) CO adsorption on top of Pt (top and side view) for Pt_2_PdPb surfaces.

The difference in the activities of Pt_2_PdPb_2_ and Pt_2_PdPb, formed by the acidic treatment of Pt_2_PdPb_2_, was studied using the DFT method. As the crystallographic structures of both ternary alloys are unknown, the structures of these alloys were constructed by modifying the known binary alloys Pb_2_Pd_3_^52^ and Pt_3_Pd.^52^ Pt_2_PdPb_2_ is formed by substituting two Pd atoms with Pt atoms within the Pb_2_Pd_3_ structure. Theoretical calculations allowed for three possibilities, but only one of them aligned with the experimental results, as indicated by the strong adsorption of DME and CO on Pt_2_PdPb; on the other hand, it was created by replacing one Pt atom with a Pb atom in the Pt_3_Pd structure. In this case, all the Pt atoms were equivalent when considering the (111) plane, resulting in the formation of only one modified surface. The XRD measurements of the pristine and acid-washed samples showed that the Pt_3_Pb/Pd_3_Pb alloy was the most abundant facet. The surfaces of Pt_2_PdPb_2_ and Pt_2_PdPb are shown in Figure 8a,d, respectively.

To understand the difference in the activities of the two catalysts, we studied the adsorption of DME and CO on each catalyst. Several adsorption sites were investigated for these molecules, and their values are summarized in Tables S7 and S8. Our study focused only on the most stable adsorption sites. The DME molecule was adsorbed through the oxygen on the top of Pb on both surfaces and the hydrogens of both methyl groups on Pd on the Pt_2_PdPb_2_ surface, whereas in the Pt_2_PdPb surface, the hydrogens of one methyl group were on Pd, and the other was on Pt, as shown in Figure 8b (Pt_2_PdPb_2_) and Figure 8e (Pt_2_PdPb). CO is adsorbed on top of the Pt atoms on the Pt_2_PdPb_2_ and Pt_2_PdPb surfaces (Figure 8c,f), in contrast to DME, which is adsorbed through different sites on both surfaces.

The adsorption energies of DME and CO on Pt_2_PdPb_2_ (pristine) and Pt_2_PdPb (acid-washed) catalysts in comparison to those of other catalyst surfaces are given in Table 4.^17,53^ Comparing the adsorption energies of DME and CO on Pt_2_PdPb with those on Pt(111) leads to the conclusion that the alloying of Pd and Pb with Pt chemically modifies the surfaces and helps weaken the adsorption of these species. Hence, DME oxidation became more facile on the Pt_2_PdPb surface because of weaker adsorption. Moreover, Pt_2_PdPb exhibited a higher tolerance to CO poisoning.

**Table 4:** Adsorption Energies of DME and CO on Pt_2_PdPb_2_ and Pt_2_PdPb

alloy	*E*_ads_ (DME) (eV)	*E*_ads_ (CO) (eV)
Pt_2_PdPb_2_	–0.90	–2.38
Pt_2_PdPb	–0.77	–1.82
Pt_3_Pd_3_Sn_2_	–0.89	–2.06
Pt3Sn	–1.03	–2.08
Pd3Sn	–0.87	–2.11
Pt(111)	–1.37	–2.13

The adsorption of DME and CO on Pt_2_PdPb is weaker (less negative) than that on Pt_2_PdPb_2_. The weaker adsorption energies enhance the electrocatalytic oxidation of DME and the removal of CO, thus reducing CO poisoning. These values explain the superior activity of Pt_2_PdPb.

#### 3.5.1 Formation of O* on the Surface

The experimental results indicated that oxygen atoms were produced on the surface of the Pt_2_PdPb alloy. The formation of these atoms was computationally studied. OH* and O* species are formed according to the reactions in eqs 11 and 12
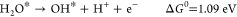
11
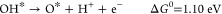
12

Both reactions are endergonic, with almost the same Δ*G*^0^ values. These reactions are potential-dependent and require a potential of 1.1 V to occur. The data for the calculated reaction energies are listed in Table S9. Each hydrogen abstraction from DME depends on the applied potential, V, leading to a decrease in the system energy by eV upon proton release.^37^ Considering that these processes occur prior to water splitting, it is anticipated that the reactions represented by eqs 11 and 12 will take place at a potential lower than 1.1 V.^37^ Moreover, Pb is an oxophilic metal; hence, water activation on Pb should occur at potentials significantly lower than those of Pt or Pd (*E*°(Pb/Pb^2+^) = −0.126 standard hydrogen electrode (SHE).

The DME oxidation mechanism has been reported previously for a Sn-based ternary metal catalyst, Pt_3_Pd_3_Sn_2_/C.^17^ Pb is very similar to Sn in many ways including the standard reduction potential of Sn (−0.126 vs −0.130 V vs SHE). Hence, the DME reaction mechanism and water dissociation processes on the acid-washed Pt_2_PdPb_2_/C catalyst occur in a manner similar to that of a Sn-based ternary catalyst. This is also evident from the similarity of DME oxidation on these two catalysts in that DME adsorbs through the interaction of oxygen on Sn (in the case of Pt_3_Pd_3_Sn_2_/C) and Pb (in the case of Pt_2_PdPb_2_/C) and the hydrogen of the methyl group to either Pt or Pd (in both catalysts). Moreover, the reaction products of DME oxidation over these two catalysts are the same. The formation of CH_3_OH, COOH, and CO_2_ was detected by online mass spectrometry analysis of DME oxidation on both catalysts. Hence, we believe that the reaction mechanism of Pt_2_PdPb_2_/C will also follow that of Pt_3_Pd_3_Sn_2_/C.

DME oxidation begins with the successive dissociation of hydrogen atoms from the methyl group, which interacts with Pt to form CH_3_OC*H. This intermediate readily undergoes C–O bond cleavage to form C*H_3_ and OC*H, which react with the OH* species, formed from water activation according to eq 11, and form CH_3_OH and CHOOH, respectively. Hydrogen scissoring from the second methyl group of CH_3_OC*H interacting with Pd can also occur, leading to the formation of C*H_2_OC*H. C–H cleavage from the CH group of C*H_2_OC*H followed by C–O bond breaking results in the formation of C*O and C*H_2_ on the Pt and Pd atoms, respectively. The adsorbed CO was oxidized by reacting with OH* to CO_2_. The reaction products (CH_3_OH and CHOOH CO_2_) were detected by mass spectroscopy.

## 4 Conclusions

A new ternary metal catalyst PtPdPb with varying metal contents prepared by a polyol method was tested for the electro-oxidation of DME. The formation of the alloys was confirmed by UV–vis spectroscopy and XRD, and phases, such as PtPd, PtPb, Pt_3_Pb, and Pd_3_Pb, were detected. A catalyst with a higher initial non-noble metal loading, Pt_2_PdPb_2_/C, was washed with the optimized HNO_3_ concentration. Elemental analysis and XPS results showed that the treatment led to the dissolution of an adequate amount of Pb but had an insignificant influence on the content of the noble metals, Pt and Pd. Following the acid treatment, a 4-fold enhancement in the catalyst performance was observed in an aqueous half-cell, which was better than the catalysts with higher initial noble metal loadings. A DDMEFC operated with DME/air (anode/cathode) using acid-washed Pt_2_PdPb_2_/C anode and Pt/C cathode catalysts of 1 and 3.1 mg_PGM_ cm^–2^ loading, respectively, provided a maximum power density of 118 mW mg_PGM_^–1^ at 70 °C. This is approximately 31% higher than the highest value reported for the Pt_0.75_Pd_0.25_/SnO_2_/C catalyst. Therefore, this study provides insight into the development of efficient postsynthesis treatment methods to further improve catalyst activity for the electro-oxidation of fuels.

## Supporting Information Available

Additional characterization data including XRD, SEM, EDS maps, EDS graph, STEM HAADF, XPS, voltammograms and fuel cell test results including crossover current density of a fuel cell fed with DME and N_2_ through the anode and cathode (PDF)
