# Supplementary material for: Chemical-Dealloying-Derived PtPdPb-Based Multimetallic Nanoparticles: Dimethyl Ether Electrocatalysis and Fuel Cell Application
Source: ACS Appl Mater Interfaces. 2023 Nov 30;15(49):56930–44. doi: 10.1021/acsami.3c11003 (PMC10726307; doi:10.1021/acsami.3c11003)
Supplement: Supplementary file 1 — am3c11003_si_001.pdf [file am3c11003_si_001.pdf]

Supporting information

Chemical Dealloying Derived PtPdPb-based Multimetallic Nanoparticles: Dimethyl Ether Electrocatalysis and Fuel Cell Application

Medhanie Gebremedhin Gebru[a], Palaniappan Subramanian[b]*, Petr Bělský[b], Radhey Shyam Yadav[a], Itay Pitussi[a], Sarath Sasi[b], Rostislav Medlín[b], Jan Minar[b], Peter Švec[c], Haya Kornweitz[a], Alex Schechter[a,b]*

[a] Department of Chemical Science, Ariel University, Ariel, 40700, Israel

[b] Research and Development Centre for Renewable Energy, New Technologies Research Centre (NTC), University of West Bohemia, Univerzitni, 8/2732 301 00, Pilsen, Czech Republic

[c] Institute of Physics, Slovak Academy of Sciences, Dúbravská cesta 9, 845 11, Bratislava, Slovak Republic

*Corresponding author email address: salex@ariel.ac.il (Prof. Alex Schechter); palans@ntc.zcu.cz (Dr. Palaniappan Subramanian)

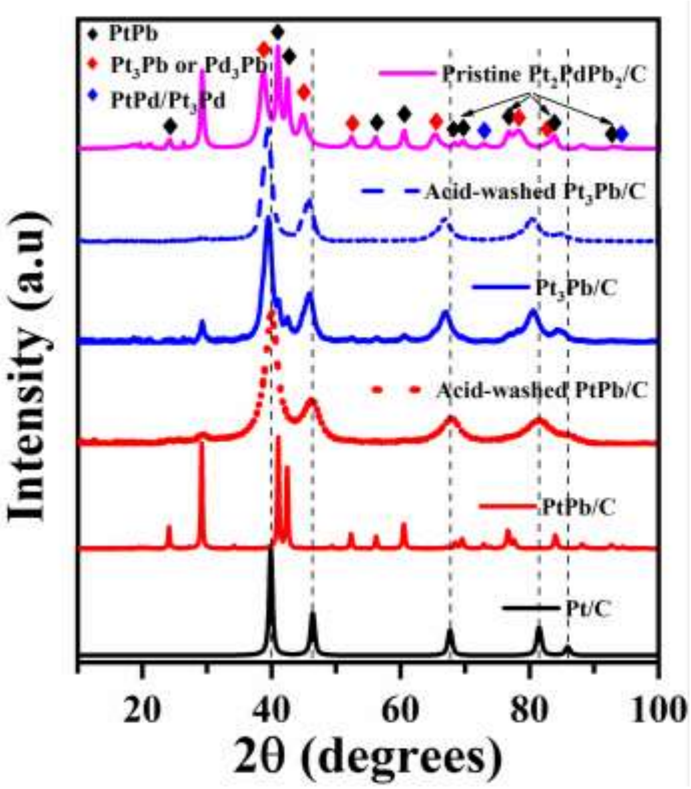

Figure S1. XRD patterns of the pristine and acid-washed (in 1 M HNO_3 for 1 h) PtPb/C and Pt_3Pb/C catalysts overlaid with Pt_2PdPb_2/C.

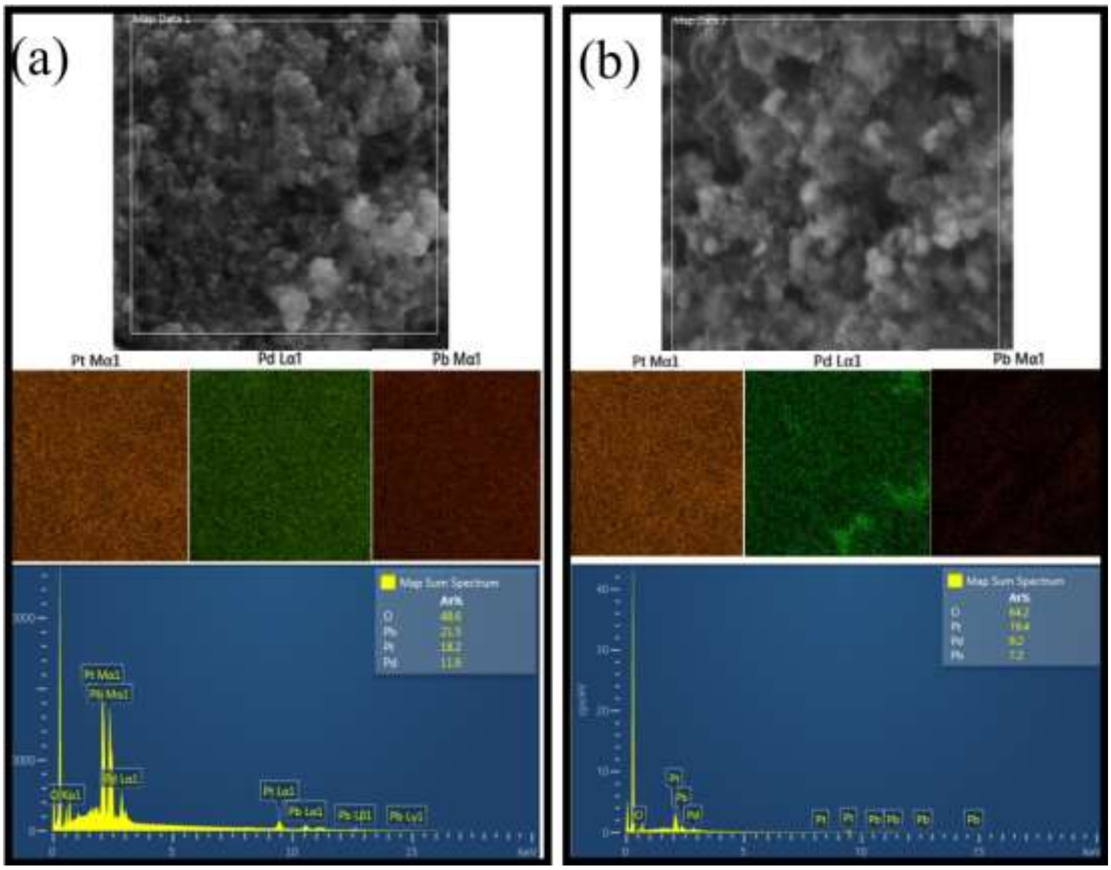

Figure S2. The SEM images, EDS mapping, and atomic ratios as measured by EDS of Pt_2PdPb_2/XC72-coated electrode (a) before and (b) after 0.6 M HNO_3 treatment of the electrode for 60 min.

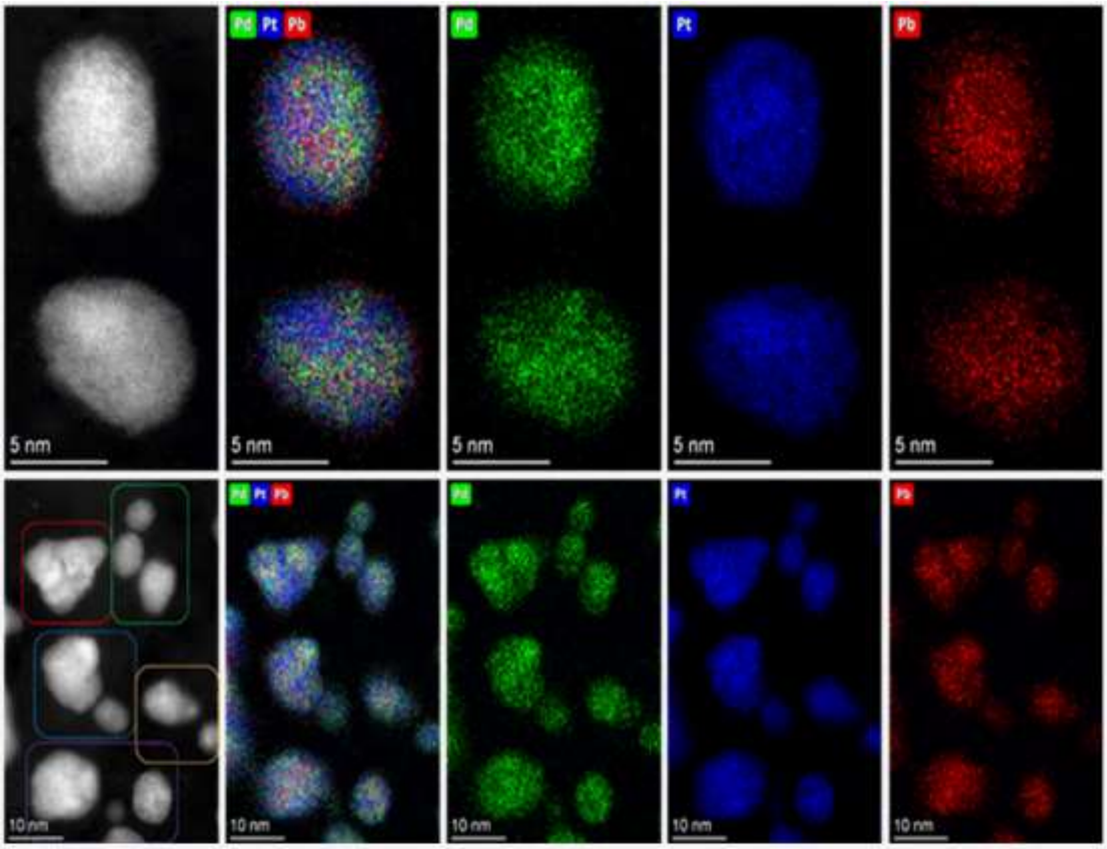

Figure S3. Typical STEM HAADF micrographs (left) and EDS maps of selected elements in pristine (top row) and acid-washed (bottom row) samples.

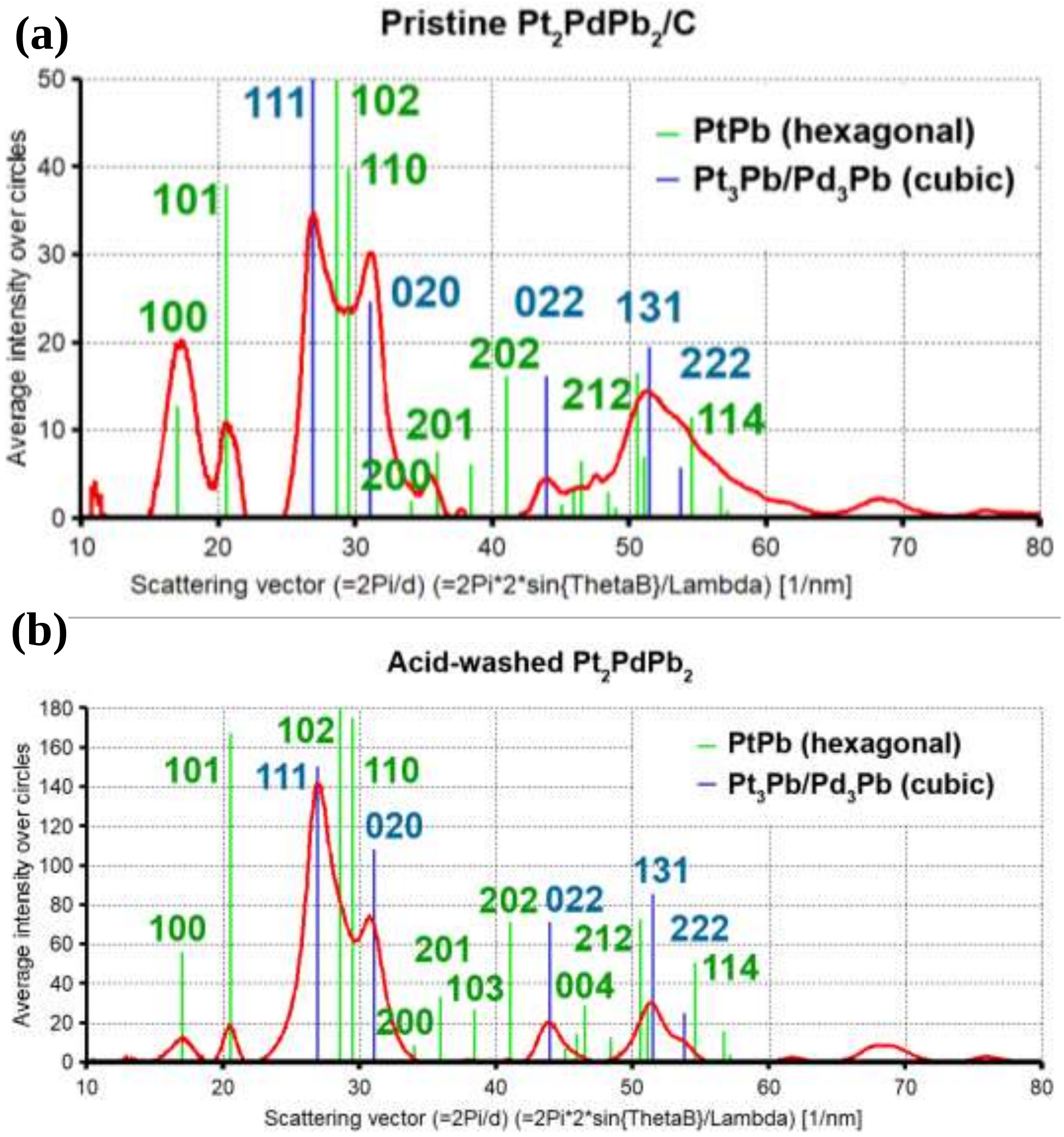

Figure S4. Processed SAED pattern of the (a) pristine and (b) acid-washed Pt_2PdPb_2/C extracted from the Indexed circular SAED patterns shown in **Figure 1C** of the manuscript.

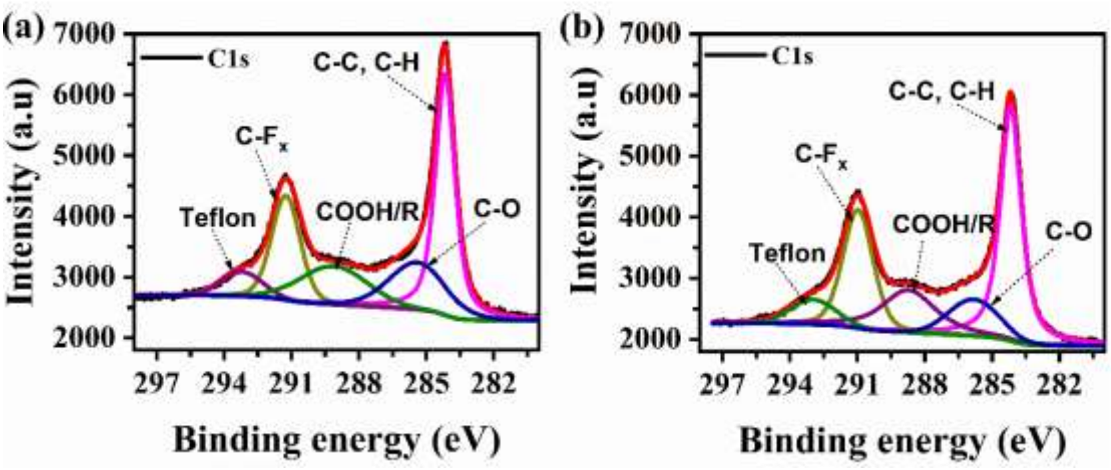

Figure S5. C1s XPS spectra of Pt_2PdPb_2/C (a) before and (b) after the catalyst acid treatment.

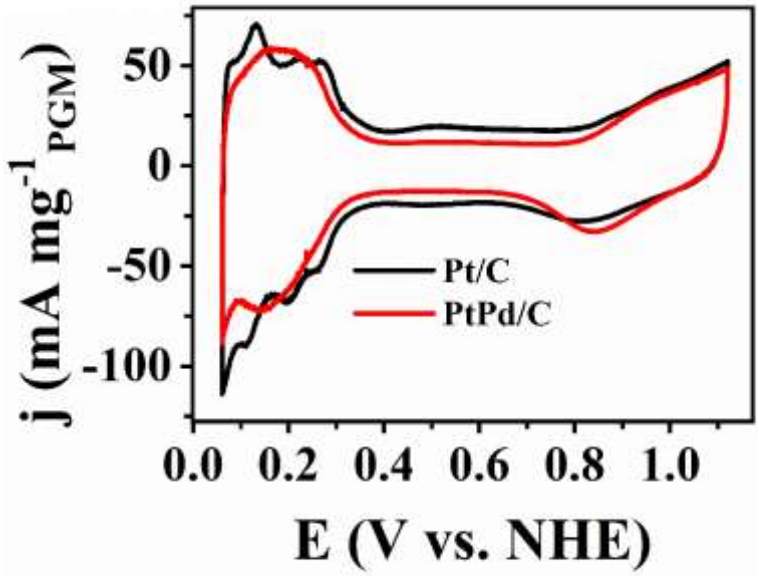

Figure S6. Comparison of CV recorded in N_2-saturated 0.5 M H_2SO_4 using Pt/C and PtPd/C (loading 50 µg cm^{-2}, scan rate 100 mV s^{-1})

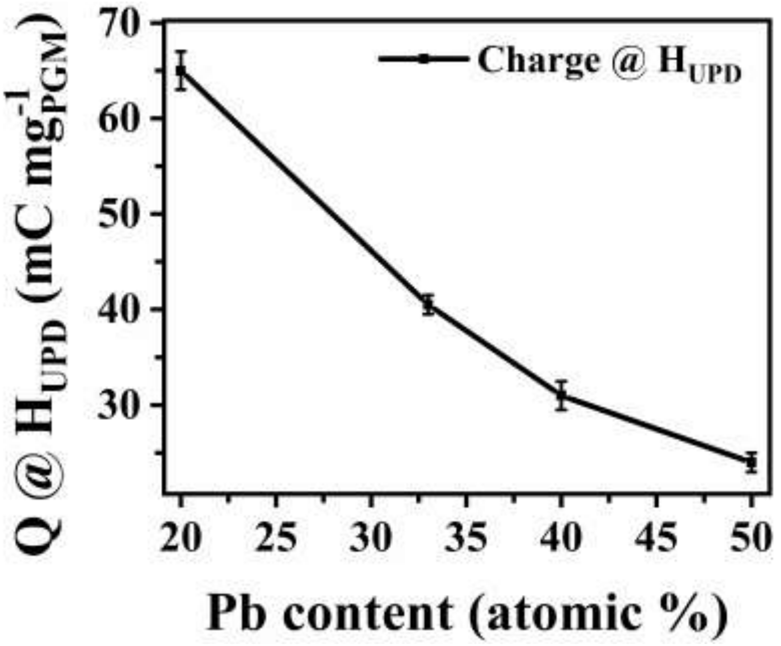

Figure S7. The change in the charge at the H_{UPD} vs. the Pb content for the CV recorded in N_2-saturated 0.5 M H_2SO_4 using $Pt_xPd_yPb_z/C$ catalysts with varying Pb content (100 mV s^{-1}). (0.5 M H_2SO_4 electrolyte, 50 µg cm^{-2} catalyst loading, scan rate 10 mV s^{-1})

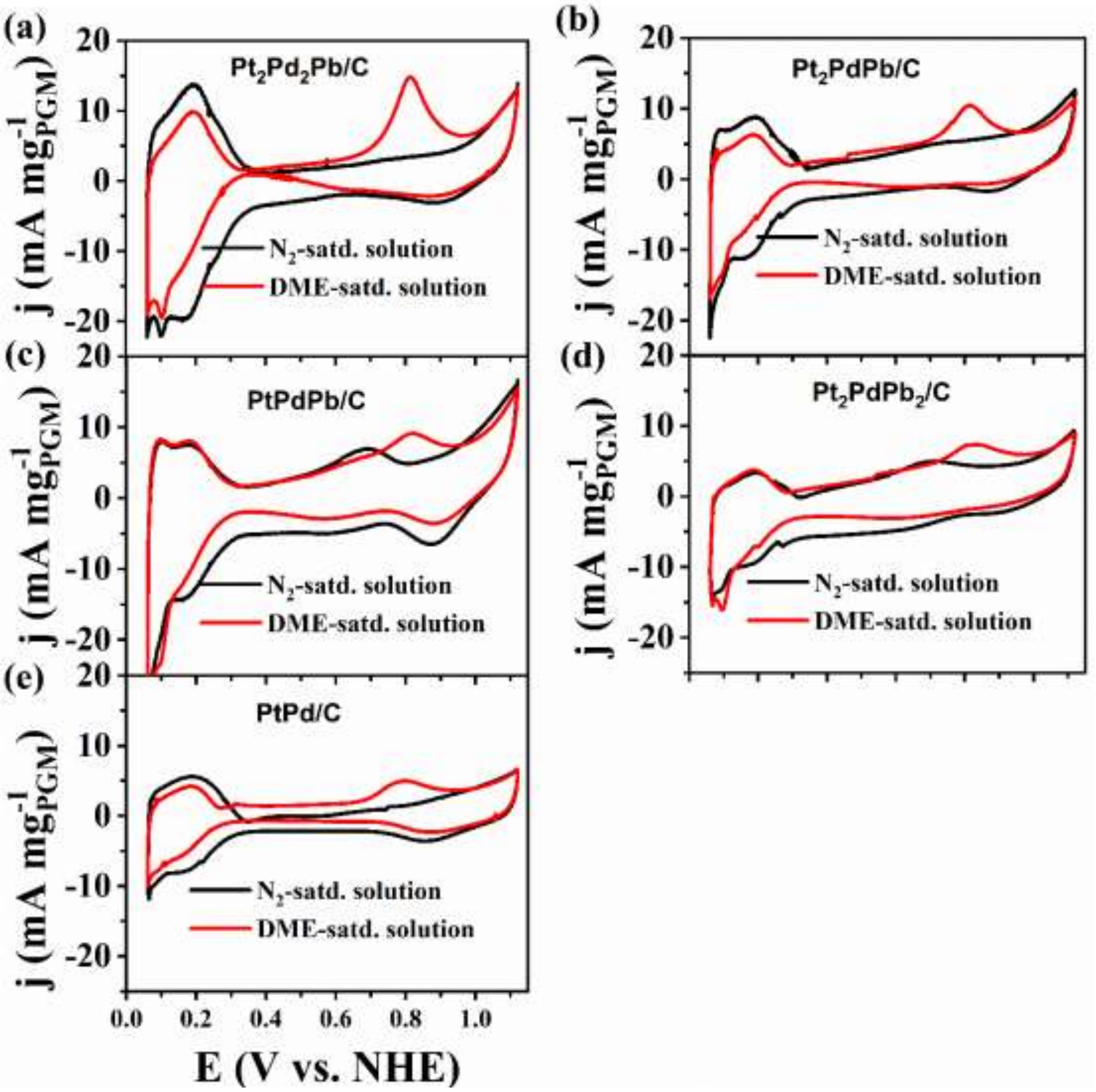

Figure S8. CV recorded in a N_2 and DME-saturated 0.5 M H_2SO_4 electrolyte solution using (a) Pt_2Pd_2Pb/C, (b) Pt_2PdPb/C, (c) PtPdPb/C, (d) Pt_2PdPb_2/C, and (e) PtPd/C catalyst-coated glassy carbon (10 mV s^{-1} scan rate, 50 μg cm^{-2} of catalyst loading)

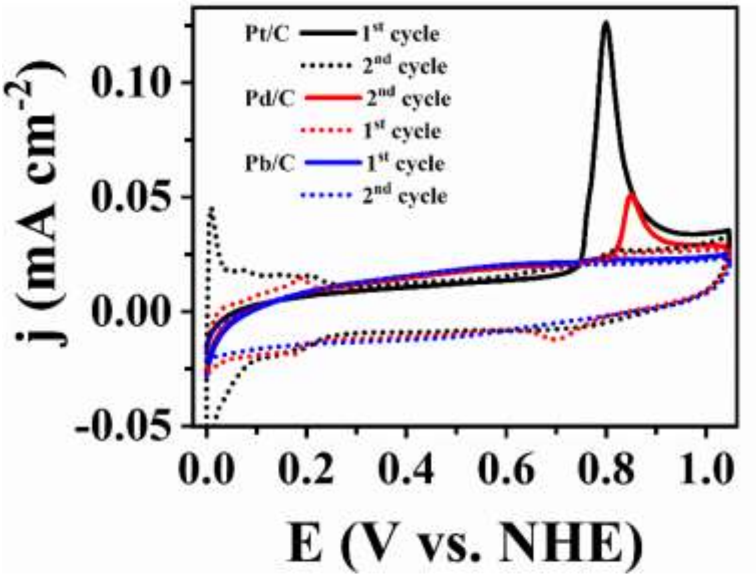

Figure S9. CO stripping measurement using Pt/C, Pd/C, and Pb/C

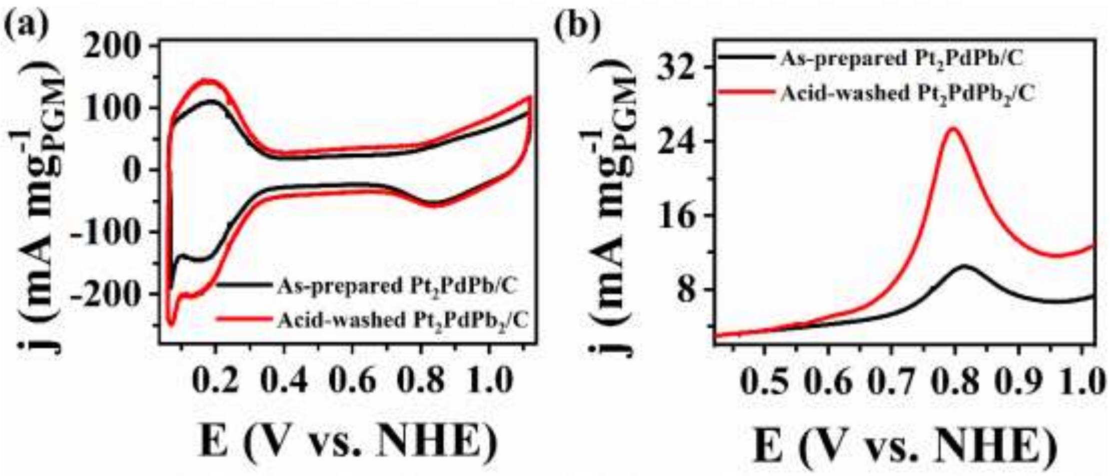

Figure S10. Comparison of voltammetric traces of Pt_2PdPb/C samples (a) CV recorded in N_2-saturated 0.5 M H_2SO_4 at 100 mV s^{-1} and (b) DME oxidation activity using 0.6 M HNO_3-washed Pt_2PdPb_2/C (~Pt_2PdPb/C) and as-prepared Pt_2PdPb/C

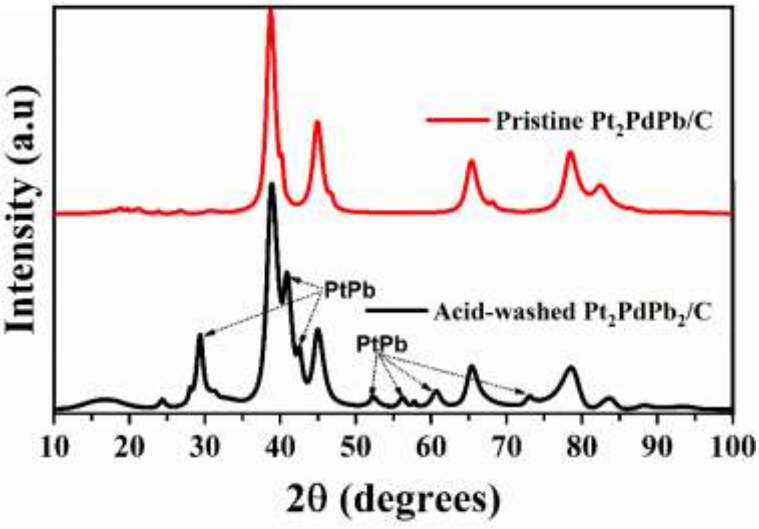

Figure S11. XRD patterns of pristine Pt2PdPb/C and 0.6 M HNO_3-washed (for 1 h) Pt_2PdPb_2/C.

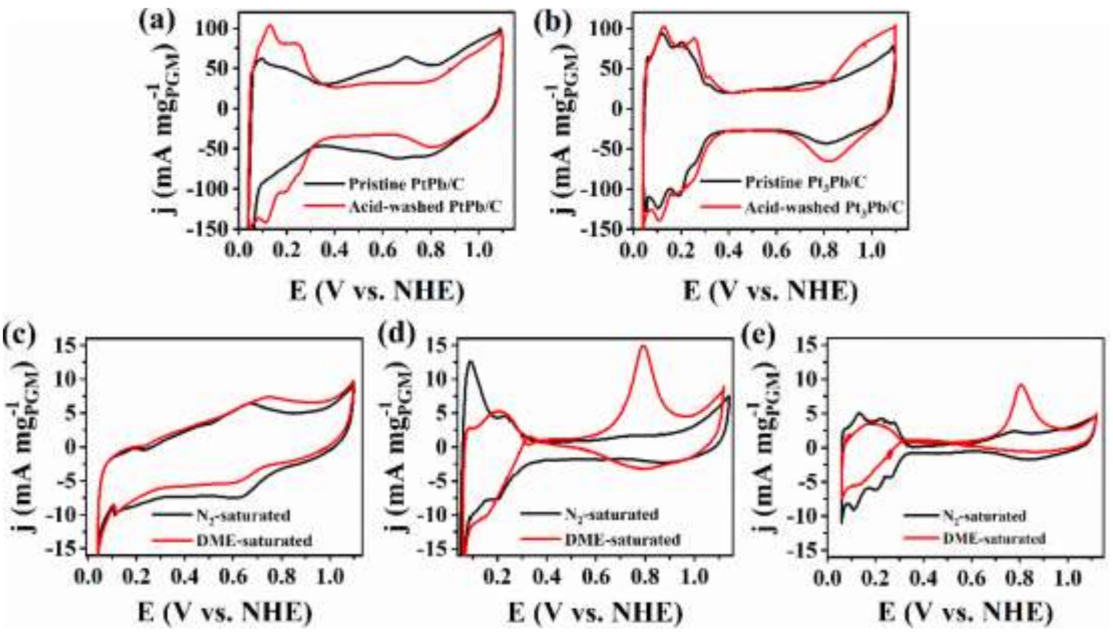

Figure S12. Voltammograms recorded in N_2-saturated 0.5 M H_2SO_4 using pristine and acid-washed (a) PtPb/C (b) Pt_3Pb/C at 100 mV s^{-1}. CV recorded in N_2 and DME-saturated 0.5 M H_2SO_4 using pristine (c) PtPb/C, (d) Pt_3Pb/C, and (e) Pt/C at 10 mV s^{-1}. (catalyst loading 50 µg cm^{-2})

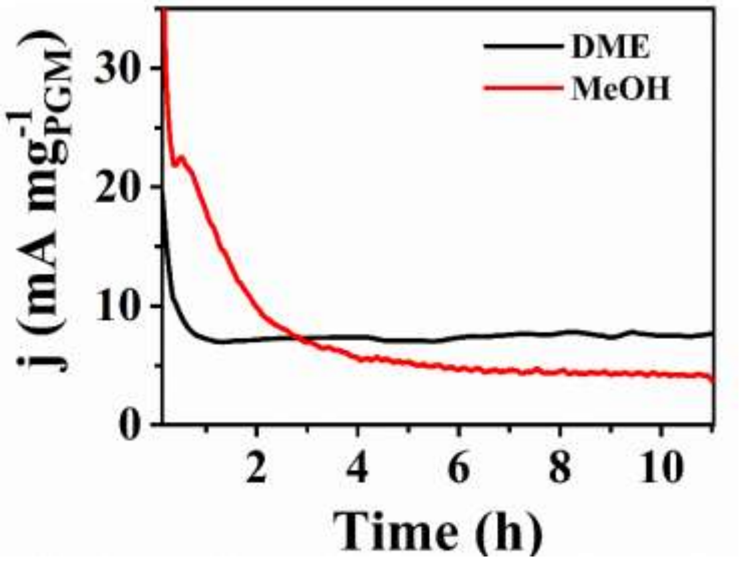

Figure S13. Current measured at 0.8 V vs. NHE using acid-washed Pt_2PdPb_2/C in an electrolyte containing equal concentrations of MeOH and DME (0.74 M) (catalyst loading 50 µg cm^{-2}; electrolyte 0.5 M H_2SO_4)

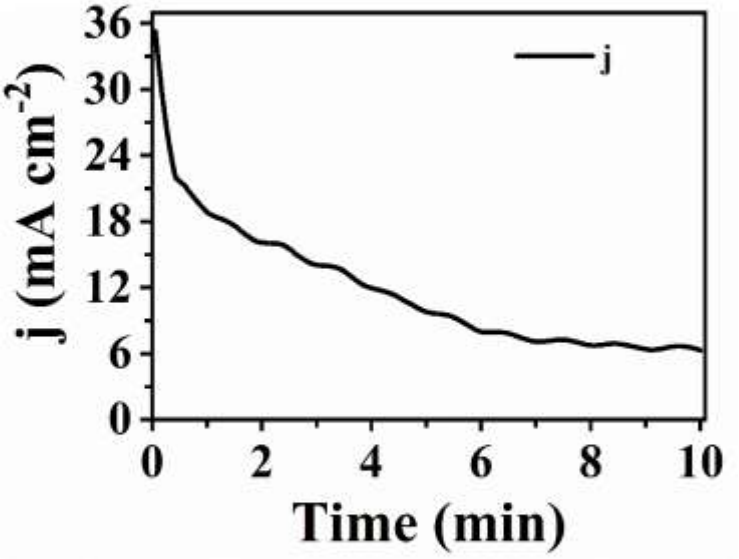

Figure S14. The crossover current measured by applying a potential of 0.8 V vs. E_{oc} on the cathode while flowing a 100% humidified DME at a flow rate of 40 mL min^{-1} through the anode and humidified N_2 at a flow rate of 10 mL min^{-1} through the cathode. The catalyst loading was 1 mg_{PGM} cm^{-2} of acid-treated Pt_2PdPb_2/C on the anode and 2.7 mg_{PGM} cm-^{2} of Pt/C on the cathode. The air flow rate was maintained at 350 mL min^{-1}, and a Nafion^{TM}212 membrane was used. The cell temperature was kept at 70 °C under ambient pressure.

Table S1. Price comparison of commonly used oxophilic metals

Element full name	Element symbol	Price ($ kg^{-1})
Tin	Sn	26.6
Bismuth	Bi	15-39
Ruthenium	Ru	19933.46
Lead	Pb	1.96

Table S2. The composition of the metals both dissolved in the solution and remained on the electrode after dipping the Pt_2PdPb_2/C-coated Toray paper in a given concentration of HNO_3 for 1 h

	ICP result (ppm)					
	Left on the electrode			Dissolved in the HNO_3 solution		
	Pt	Pd	Pb	Pt	Pd	Pb
Untreated electrode	2.996 ± 0.52	0.798 ± 0.17	3.174 ± 0.63	<0.005 ± 0.00	<0.005 ± 0.00	<0.004 ± 0.00
0.3 M HNO_3	2.979 ± 0.33	0.803 ± 0.11	2.938 ± 0.09	<0.005 ± 0.00	<0.005 ± 0.00	0.313 ± 0.04
0.6 M HNO_3	2.949 ± 0.14	0.792 ± 0.23	1.675 ± 0.20	<0.005 ± 0.00	0.012 ± 0.00	1.573 ± 0.33
1.2 M HNO_3	2.757 ± 0.27	0.775 ± 0.15	1.560 ± 0.12	0.302 ± 0.03	0.026 ± 0.01	1.702 ± 0.24

Table S3. EDS compositional results for marked individual particles in **Figure S3**. Pristine top, acid-washed bottom.

**Pristine**		**Particle top**		**Particle bottom**	
Element	Family	At. %	At. %, Error	At. %	At. %, Error
Pd	L	22.70	1.72	22.08	1.71
Pt	L	56.19	2.99	59.02	2.92
Pb	L	21.11	2.23	18.90	2.07

**Acid washed**		**Red group**		**Green group**		**Blue group**		**Orange group**		**Purple group**	
Element	Family	At. %	At. % Error	At. %	At. % Error	At. %	At. % Error	At. %	At. % Error	At. %	At. % Error
Pd	L	19.87	1.63	21.88	1.74	20.28	1.63	22.75	1.78	20.98	1.66
Pt	L	64.87	2.73	62.39	2.80	62.79	2.82	61.05	2.84	61.16	2.87
Pb	L	15.26	1.77	15.73	1.80	16.92	1.92	16.20	1.83	17.86	1.99

Table S4. The atomic percentage of Pt, Pd, Pb, and their oxides extracted from the XPS spectra shown in **Figure 3**.

Pt_2PdPb_2 before acid wash						
Metals	Peak areas					Sum of peak areas
	Peak1	Peak2	Peak3	Peak4	Peak5	
Pt	1990.528	423.285	3041.096			5454.909
Pd	1359.473	40.936	727.137			2127.546
Pb	2121.550	3162.414	1776.682	2506.669		9567.315
C	5902.200	2409.480	1769.920	3997.650	1090.110	15169.360
Pt_2PdPb_2 after acid wash						
Metals	Peak areas					Sum of peak areas
	Peak1	Peak2	Peak3	Peak4	Peak5	
Pt	2017.232	455.869	3529.209			6002.310
Pd	837.051	382.560	617.724	443.784		2281.119
Pb	897.639	1780.819	787.190	1664.822		5132.480
C	5462.84	2397.65	1076.84	4775.910	746.990	14460.230

Note: peaks are numbered in increasing the binding energy of each metal

Table S5. Atomic ratios of the synthesized catalysts as measured by ICP

S. No.	Catalyst	Atomic ratio		
		Pt	Pd	Pb
1	PtPb/XC72	1.01 ± 0.08	0.03 ± 0.02	1.00 ± 0.00
2	PtPd/XC72	0.97 ± 0.04	1.00 ± 0.13	0.00 ± 0.00
3	PtPdPb/XC72	1.00 ± 0.11	1.01 ± 0.14	1.00 ± 0.00
4	Pt_2PdPb/XC72	1.91 ± 0.05	1.00 ± 0.00	1.00 ± 0.00
5	Pt_2Pd_2Pb/XC72	1.94 ± 0.12	1.99 ± 0.16	1.00 ± 0.00
6	Pt_2PdPb_2/XC72	0.97 ± 0.14	0.50 ± 0.07	1.00 ± 0.00

Table. S6. Summary of electrochemical parameters of Pt_2PdPb_2/XC72 treated at varying HNO_3 concentrations towards DME oxidation

HNO_3 concentration	j_p	Q_{oxi}	Onset potential
	mA mg_{PGM}^{-1}	mC mg_{PGM}^{-1}	V, vs. NHE
untreated electrode	2.53 ± 0.01	15.97 ± 0.19	0.50 ± 0.01
0.15 M	3.51 ± 0.00	53.33 ± 0.16	0.51 ± 0.00
0.3 M	4.19 ± 0.19	54.27 ± 0.96	0.51 ± 0.01
0.6 M	5.10 ± 0.11	65.51 ± 1.46	0.52 ± 0.00
0.9 M	4.86 ± 0.00	58.75 ± 0.00	0.50 ± 0.00
1.2 M	4.45 ± 0.15	47.94 ± 0.00	0.51 ± 0.01

Table S7. Adsorption energy of DME for various possible configurations.

Catalyst	Adsorption site	Adsorption	E_{ads} (eV)
	Hollow (Pd_Pt_Pt)	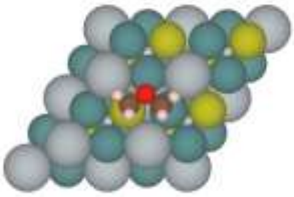	-0.61
	Hollow (Pd_Pt_Pb)	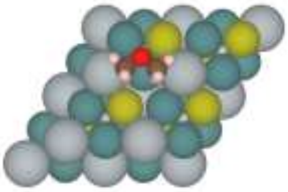	-0.49
	Hollow (Pt_Pd_Pb)	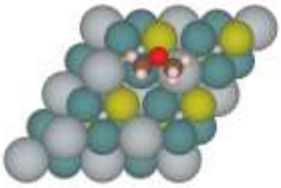	-0.51
**Pt_2PdPb**	Hollow (Pt_Pb_Pd)	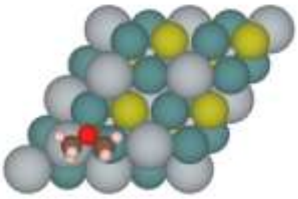	-0.50
	Hollow (Pb_Pt_Pt)	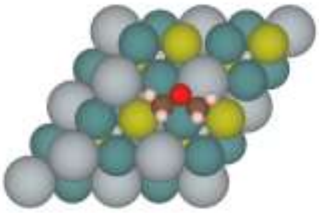	-0.69
	Bridge (Pt_Pd-O)	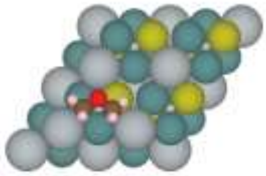	-0.60

	Bridge (Pd_Pt-O)	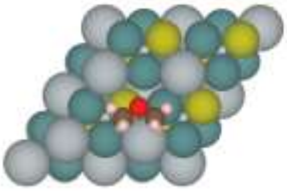	-0.53
	Bridge (Pb_Pt-O)	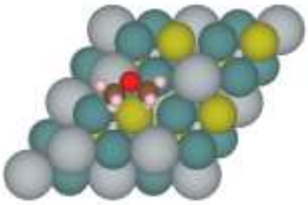	-0.74
	Top (Pb-O)	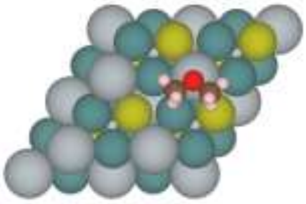	-0.75
	Top (Pt-O)	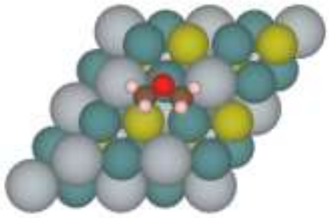	-0.62
	Top (Pd-O)	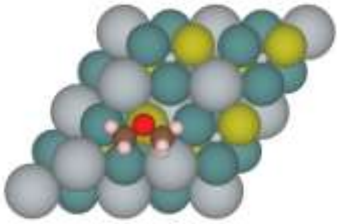	-0.57
	**Top (Pb-O)**	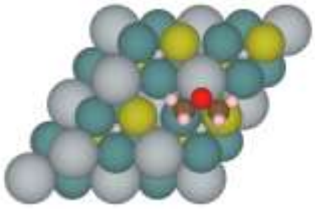	**-0.77**
**Pt_3Sn (111)**	Top (Sn)	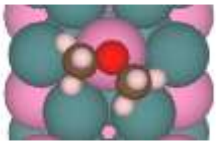	**-1.03**

	Top (Pt)	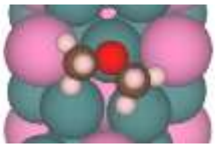	-0.75
	Top (Pd)	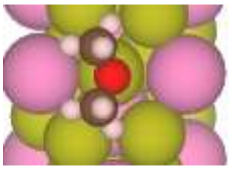	**-0.87**
**Pd_3Sn (111)**	Top (Pd)	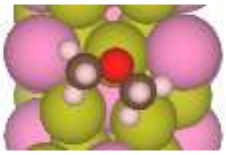	-0.80
	Top (Sn)	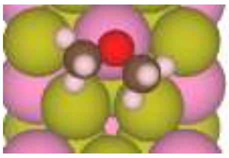	-0.63
**Pt (111)**	Top (Pt)	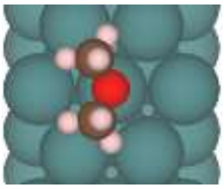	**-1.37**

Table S8. Adsorption energy of CO for various possible configurations.

Catalyst	Adsorption site	Adsorption	E_{ads} (eV)
	**Top (Pt)**	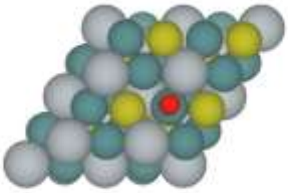	**-1.82**
**Pt_2PdPb**	Top (Pd)	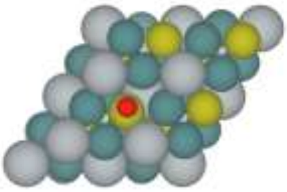	-1.38
	Top (Pb)	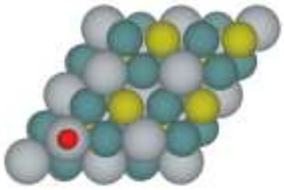	-0.08
	Top (Pt)	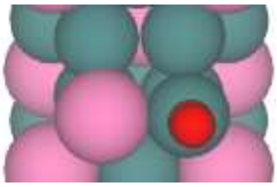	-1.95
**Pt_3Sn (111)**	**Hollow**	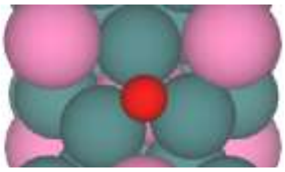	**-2.08**
	Bridge (Pt-Pt)	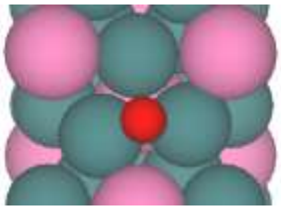	-1.99
**Pd_3Sn (111)**	Top (Pd)	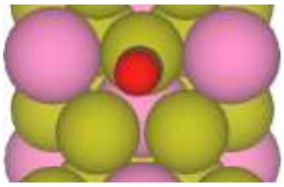	-1.47

	**Hollow**	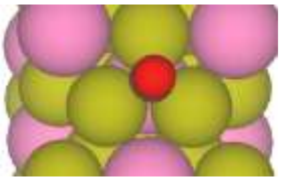	**-2.11**
	Bridge (Pd-Pd)	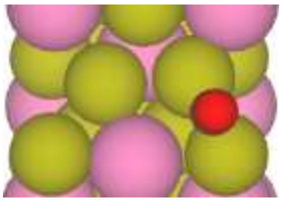	-1.47
	Top (Pt)	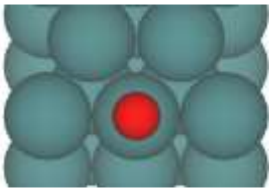	-2.06
**Pt (111)**	**Hollow**	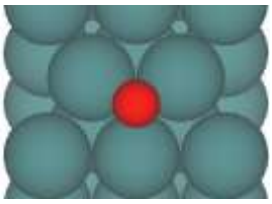	**-2.14**
	Bridge (Pt-Pt)	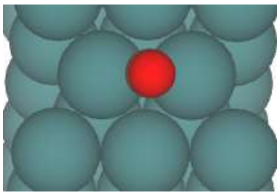	-2.04

Table S9. DFT calculated energy (eV) with ZPE correction used in equations (11) and (12) in the main manuscript.

**Species*/Pt_2PdPb**	**DFT calculated energy (eV) (with ZPE correction)**
H_2O	-504.60
OH	-500.22
O	-495.83
H_2 (aq)	-6.58
